# Constructing Physiological Defense Systems against Infectious Disease with Metal–Organic Frameworks: A Review

**DOI:** 10.1021/acsabm.3c00391

**Published:** 2023-08-10

**Authors:** Nikita O. Mishra, Alisa S. Quon, Anna Nguyen, Edgar K. Papazyan, Yajiao Hao, Yangyang Liu

**Affiliations:** † Department of Chemistry and Biochemistry, California State University, Los Angeles, 5151 State University Drive, Los Angeles, California 90032, United States

**Keywords:** metal−organic frameworks, infectious disease, vaccines, macromolecule protection, antiviral, antibacterial, drug delivery, personal protective equipment

## Abstract

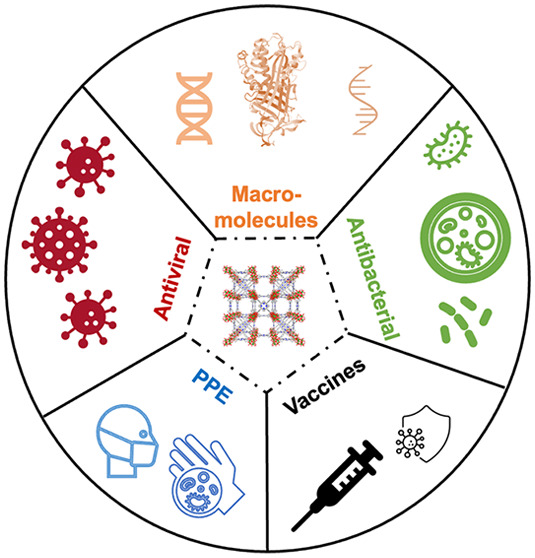

The swift and deadly spread of infectious diseases, alongside the rapid advancement of scientific technology in the past several centuries, has led to the invention of various methods for protecting people from infection. In recent years, a class of crystalline porous materials, metal–organic frameworks (MOFs), has shown great potential in constructing defense systems against infectious diseases. This review addresses current approaches to combating infectious diseases through the utilization of MOFs in vaccine development, antiviral and antibacterial treatment, and personal protective equipment (PPE). Along with an updated account of MOFs used for designing defense systems against infectious diseases, directions are also suggested for expanding avenues of current MOF research to develop more effective approaches and tools to prevent the widespread nature of infectious diseases.

## 1 Introduction

Infectious diseases are a major global health issue, causing millions of deaths across the globe annually. The development of vaccine technology and other defense systems against viral and bacterial pathogens has allowed for the prevention of many infectious diseases, and this field is still rapidly expanding. Traditional vaccine technologies include live attenuated vaccines, toxoids, and inactivated pathogens.^1,2^ The recent onset of severe acute respiratory syndrome coronavirus 2 (SARS-CoV-2) led to the rapid development of different types of vaccine technology using biomolecules like ribonucleic acid (RNA), deoxyribonucleic acid (DNA), and proteins. A need has arisen to protect these biomolecules as they travel through the body and deliver them to the target cells. In addition, new and more effective therapeutic approaches are urgently needed to treat infections caused by antibiotic-resistant bacteria or rapidly mutating viruses, such as the coronavirus.

COVID-19 prevention and treatment have been of particular interest due to the onset of the pandemic caused by SARS-CoV-2 in 2019. COVID-19 vaccines underwent rapid development, with the first rounds of vaccination in the United States occurring just over a year after the pandemic began.^3^ Although the vaccine distribution was largely successful, the requirements for cold-chain storage and transportation can be a major hurdle for its distribution in many developing countries. Most of the issues faced by COVID-19 vaccines could also be extended to other vaccines, such as those for measles, mumps, rubella, and varicella.^4^ Therefore, there is an urgent need to develop new methods to effectively protect vaccines from degradation without adding a high cost.

Furthermore, the rapid-evolving nature of pathogens like viruses and bacteria has also brought about a need for novel antiviral and antibacterial compounds. Challenges in antibacterial drug development include improving drug efficacy and selecting targets in the bacterial genome that would be least susceptible to rapid resistance.^5^ There have been similar issues in antiviral drug development, with a specific focus on improving the efficacy of drugs to avoid outbreaks that could potentially cause pandemics.^6^

One effective tool for fighting infectious diseases during the COVID-19 pandemic was personal protective equipment (PPE). Developing PPE that can effectively defend against infectious diseases is particularly important for frontline workers; however, much remains to be done. Many existing filters and fabrics do not work for extended periods, especially considering that some pathogens can “stick” to such textiles several days after initial exposure.^7^ In addition, the mass production of some of these materials has caused environmental issues.^8^ Moreover, some of the most effective protective clothing and equipment, such as full-body suits accompanied by respirators, can provide better protection but at the cost of mobility and dexterity.^9^

Each of the issues discussed above presents its own challenges and needs; fortunately, the discovery and development of new materials, such as metal–organic frameworks (MOFs) and their derivatives, have provided potential solutions to some of these problems. MOFs are a novel class of crystalline porous materials that consist of metal ions/clusters and organic linkers. The countless metal–ligand combinations and diverse functional groups can give rise to a large variety of MOFs that may be suitable for a broad range of applications, including gas storage,^10−12^ separation,^13,14^ catalysis,^15,16^ chemical sensing,^17,18^ solar cells,^19^ drug delivery,^20−22^ and other biomedical applications. Compared to other porous materials, MOFs have several advantages that make them outstanding candidates for biomedical applications, including their (i) high surface areas allowing high drug/guest loading, (ii) large pores that can accommodate various guest (macro)molecules, (iii) tunable structure, functionality, and particle size unlocking various materials design, (iv) excellent stability offering protection to encapsulated cargo, and (v) unique pH-responsiveness enabling the target delivery to affected cells.

**Figure 1:**
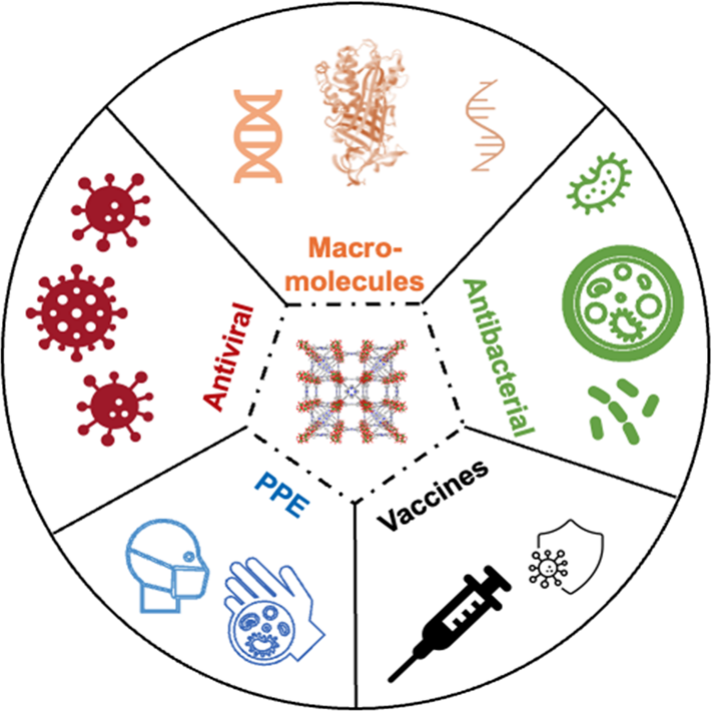
An overview of MOF applications discussed in this paper.

In recent years, the rapid research development in MOFs has led to numerous structures with tunable properties, enabling their applications in fighting infectious diseases. This review provides an overview of MOFs used in constructing physiological defense systems against infectious diseases, including those studied for macromolecule/vaccine delivery, antiviral/antibacterial treatments, and PPE (**Figure 1**). In addition, this review summarizes how MOFs could be utilized to improve therapeutic efficacy and create more effective prevention and treatment methods for infectious diseases by highlighting recent advances on this topic.

## 2 MOF Selection and Consideration

Various factors must be considered when using MOFs for the prevention and treatment of infectious diseases, such as their biocompatibility, cytotoxicity, stability under physiological conditions, and specific structural features. Biocompatibility is often described by the stability of the MOF in the bloodstream (or more broadly, in buffers of pH ∼7.4) and how the MOF interacts with its surroundings based on size, surface, and charge. While metals like chromium (Cr) were initially studied for their biomedical uses, the focus was later shifted to less toxic metals like iron (Fe), zinc (Zn), and zirconium (Zr).^23^ A study on lethal dosing in rats showed that calcium (Ca), magnesium (Mg), titanium (Ti), Zn, Fe, and Zr are most suitable when designing biocompatible MOFs.^24^ The organic linker is another important aspect to consider; if the linker compound is not naturally found *in vivo*, it must be able to be absorbed by the body, which can often be fine-tuned by adding functional groups, such as amino, nitro, carboxylate, etc. In addition, such linkers must be secreted without causing damage to organ systems, especially the kidney.^25^ Using amino-acid-based linkers or other endogenous molecules may be more suitable when designing biocompatible MOFs, as it decreases the risk of malabsorption or toxicity. However, the use of these molecules as linkers is limited due to low stability and porosity.^26^ Most biocompatible MOFs reported to date are constructed from carboxylate-based and imidazolate linkers due to their low toxicity (**Table 1** and Table S1).

**Table 1:**
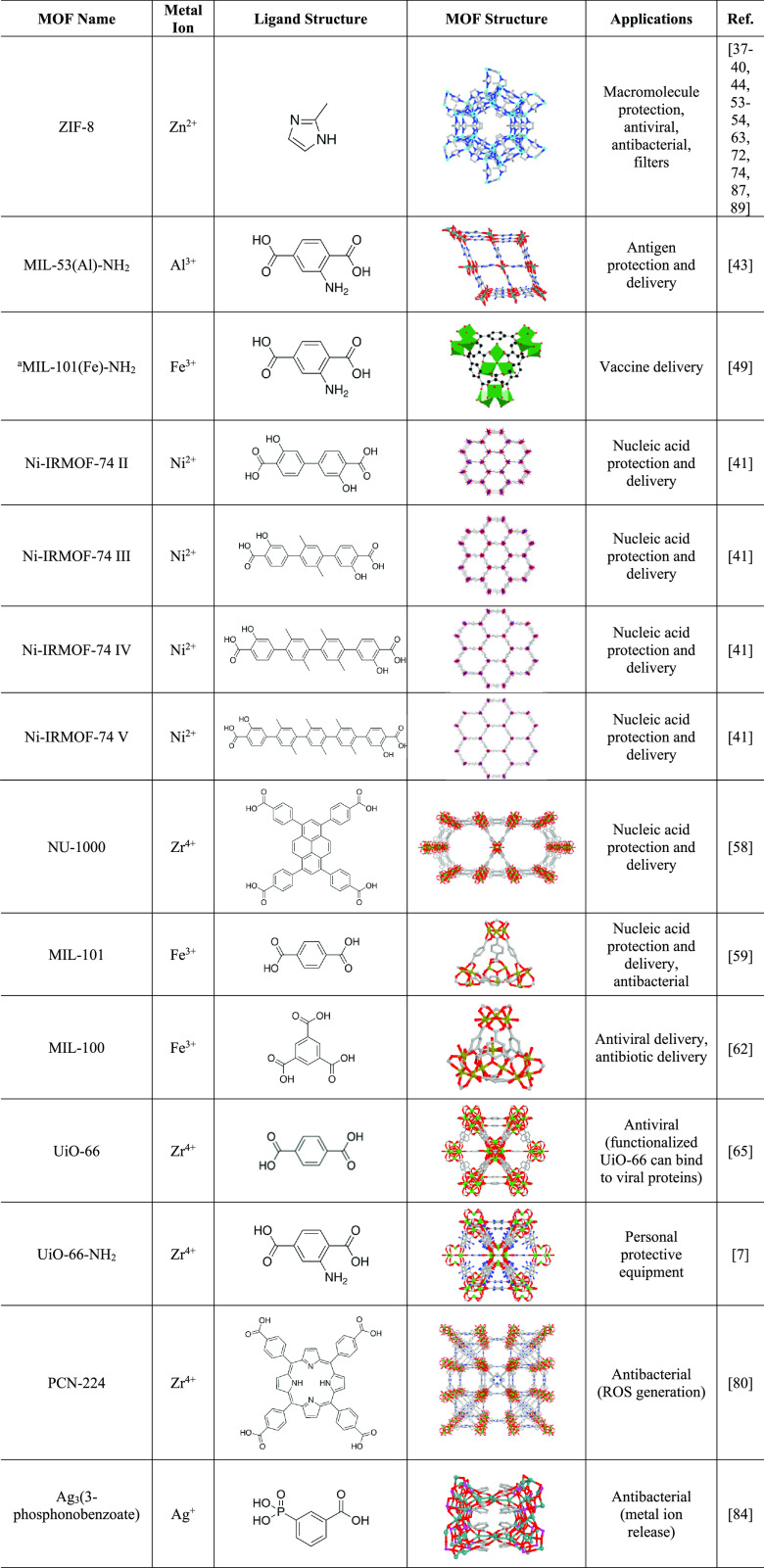
Names, Metal Ions, Ligands, and Structures of the MOFs and Their Specific Applications Related to the Defense and Treatment of Infectious Diseases As Discussed in This Review

a The crystal structure shown does not include −NH_2_.

The crystalline/particle size of MOFs is another important factor to consider. Outstanding physicochemical properties were observed for nanoparticles (1–100 nm) due to their enhanced permeability.^27,28^ The synthesis of most MOFs can be fine-tuned to produce nanoparticles, serving as nanomedicine or nanocarriers for therapeutics.^29,30^ Other factors to consider when selecting a MOF for biological/biomedical applications are their ease of production and specificity. Studies showed that some MOFs can degrade in buffers of a specific pH and subsequently release the loaded cargo (drug or biomolecules), exhibiting pH-responsiveness.^31,32^ These pH-responsive MOFs can achieve targeted/selective delivery of biomolecules/drugs to specific cells, improving efficiency and minimizing side effects. In addition, modifying the MOF surfaces with polymers was shown to improve the chemical stability of MOFs, allowing the fine-tuning of their cargo release properties in different biological environments. For example, Forgan et al. modified the surfaces of UiO-66 nanoparticles with polyethylene glycol (PEG) and observed enhanced MOF stability in phosphate-buffered saline (PBS) and improved pH responsiveness.^33^ This surface modification also enhanced caveolae-mediated endocytosis, allowing higher cell uptake of the nanoMOF and the encapsulated cargo.

There are three primary methods by which MOFs can be loaded with cargo (which may include biomolecules or antiviral/antibacterial compounds): encapsulation, direct assembly, and postsynthetic methods. Encapsulation takes advantage of noncovalent interactions that allow cargo to settle into MOF pores without changing the structure of the framework. In direct assembly, the cargo is built into the framework of the MOF and usually serves as a part of the ligands that connect the metal ions together. Postsynthetic strategies attach cargo molecules to the surface either through coordination or covalent bonding to the MOF or via adsorption onto MOF surfaces (which is often due to weaker interactions such as hydrogen bonding or π stacking).^20^ It is important to consider which of these strategies is best suited for the cargo of interest when designing MOF-based systems.

To date, various MOFs have been explored for applications related to infectious disease defense and treatment, and many of these MOFs are biocompatible with minimal cytotoxicity.^23,24,34−36^**Table 1** outlines the structures of the MOFs discussed in this review and their specific applications related to the defense and treatment of infectious diseases. We also summarized other MOFs reported to date for related applications in Table S1. Among the reported MOFs, zeolitic imidazolate framework-8 (ZIF-8) (**Table 1**), which contains biocompatible zinc clusters and imidazole linkers, has been extensively studied for biomedical applications because of its unique properties.^37−40^ First, most ZIFs can be synthesized under mild conditions at room temperature, preventing biomacromolecule degradation and allowing for ideal encapsulation conditions. In addition, ZIF-8 is stable under physiological conditions, and the organic linkers in ZIF-8 can dissociate under mildly acidic conditions.^37^ Such characteristics make ZIF-8 a perfect carrier for active therapeutics or biomacromolecules: ZIF-8 not only can encapsulate and protect these guest molecules as they travel to target cells but also the framework can degrade under the acidic conditions of the lysosome and the endosome to release the guest molecules upon uptake.

In addition to ZIF-8, other MOFs constructed from aluminum (Al), Fe, Zr, or nickel (Ni) metal nodes and various carboxylate-based organic linkers have also been studied for their applications against infectious diseases. Aluminum is a generally safe choice of metal due to its low toxicity in humans, and aluminum oxyhydroxide nanoparticles were used to improve antigen immune response.^34^ In addition, iron-based MOFs are a popular choice for biomedical applications due to their biostability and low toxicity *in vivo*.^39^ Furthermore, zirconium MOFs have generally been demonstrated as excellent candidates for drug delivery due to their stability toward hydrolysis.^40^ Lastly, the Ni-IRMOF-74 series exhibited remarkable stability in pH 3 to 11 solutions, making them excellent materials for biomedical applications in various environments and cell types.^41^

## 3 Macromolecule Protection for Vaccines

Live-attenuated vaccines long-served as a standard for creating vaccines. After the polio vaccine used a dead virus to stave off infection, many leaps have been made in the scientific community to use different macromolecules, proteins, nucleic acids, etc., as the basis for vaccination.^1^ One broad aim of vaccine development is to ensure that the antigen/adjuvant systems reach immune cells such that they can elicit an appropriate immune response before degradation. With the expanding number of macromolecules being used to create vaccines, there is a need for physiologically safe materials that can be used to protect and stabilize these macromolecules to improve the stability and target delivery of vaccines.

### 3.1 Vaccine and Antigen Protection

The unique properties of MOFs make them ideal materials for vaccine delivery. First, nanoparticulate MOF delivery platforms can create a positive immune response by allowing efficient codelivery of antigens and adjuvants to immune cells, leading to an overall increase in long-term immunity.^42^ In addition, the unique pH-responsive properties of some MOFs enable the precise delivery of antigens/adjuvants to targeted cells, minimizing off-target release and enhancing vaccine efficacy. Furthermore, MOF carriers can serve as an “armor” around the vaccine molecules to improve their stability, allowing some vaccines to be administered orally.^43^

For example, Zhang’s group used MOF nanoparticles to create a MOF-based vaccine with enhanced efficacy.^24^ In their study, ovalbumin (OVA) was encapsulated in ZIF-8 nanoparticles followed by surface attachment of unmethylated cytosine-phosphate-guanine oligodeoxynucleotides (CpG ODN) through electrostatic interaction, affording the OVA@ZIF-8-CpG system. The encapsulation, release, and immune response of the antigen/adjuvant system were tested, showing that OVA@ZIF-8-CpG induced a stronger immune response than a simple mixture of OVA, CpG, and ZIF-8. The enhanced performance of the MOF-based vaccine was attributed to the MOF-enabled controllable pH-responsive release and delivery of both adjuvant and antigen into the same antigen-presenting cell (APC).

Utilizing the pH-responsiveness of ZIF-8, Li et al. developed cancer vaccines using MOF-gated mesoporous silica (MS@MOF) to facilitate the target delivery of the antigen and immunopotentiator, creating lasting tumor-suppressor effects with a lower dosage.^44^ The MOF acted as a gatekeeper within the delivery system, protecting the incorporated antigen and immunopotentiator to avoid the prerelease of the antigen/adjuvant system. The pH-responsiveness of ZIF-8 coating led to a slow release in a neutral environment but a fast release in an acidic environment, allowing target delivery of the antigen/adjuvant to the APCs. This example shows that the target delivery of immunology-associated large molecules can be achieved using pH-responsive MOFs, enhancing the effectiveness of vaccines *in vivo*.

Many MOFs are known to be very stable under physiological conditions, making them suitable to protect vaccines in their structural forms until being ingested by cells.^39^ Oral vaccines are highly desirable due to their ease of use and ability to induce complete immune responses (both systemic and mucosal). However, several issues arise with direct gastrointestinal (GI) delivery: antigens can be easily degraded in the GI environment, and it has proven extremely difficult to induce high cellular uptake by microfold cells in the GI mucosal membrane and increase antigenic activity. To solve these problems, Miao et al. constructed a delivery system using OVA@Al-MOF to protect antigens as they travel through the GI tract and used yeast cells to assist with the crossing of the mucosal membrane; this MOF is structurally analogous to MIL-53(Al)-NH_2_.^43^ In the delivery system, the Al-MOF formed a positively charged cage around the antigen that resisted the harsh conditions of the GI tract and showed sustained antigenic release after the intracellular vaccine uptake assisted by yeast cells. Such a system produced high levels of systemic and mucosal immune antibodies, leading to long-lasting immunity. This work demonstrated that MOFs could assist vaccine delivery by acting as “armor” around a biomacromolecule as it travels through various physiological environments.

Overall, MOFs have shown great potential in their usage as vaccine delivery vehicles, protecting and delivering both antigens and adjuvants. Although there have only been a few studies on the vaccine and antigenic delivery using MOFs, the recent development in vaccine technologies to combat the coronavirus pandemic has brought the need to enhance the protection and cellular uptake of mRNA, DNA, and proteins. As discussed in the following sections, MOFs are also excellent materials for protecting and delivering all such biomacromolecules.

### 3.2 Protein Protection and Delivery

Direct delivery of proteins has been of great interest because of the high specificity and few side effects.^33,45^ Current protein delivery systems often incorporate proteins via adsorption or surface conjugation, which can circumvent the short half-lives of proteins. However, many of these systems suffer from low target protein uptake and decreased protein activity.^46,47^ In contrast, MOFs with open pore structures can improve the efficacy of protein therapeutics by encapsulating a high load of protein inside.^48,49^ Cheng et al. used ZIF-8 to load proteins through biomineralization between the metal, ligand, and protein.^48^ The nanoparticulate system showed 94% loading efficiency, nearly 50 times the loading content of surface conjugation delivery systems. The high loading efficiency of the MOF system was attributed to the high surface area of ZIF-8 and its noncovalent interactions with the protein. Cheng et al. also found that the ZIF-8-loaded proteins retained their activity after encapsulation. This work demonstrated that placing protein subunits in MOFs can protect proteins from proteolytic degradation while maintaining the protein structure and function.

Using MOFs as the delivery vessel can also improve the efficacy of protein vaccines. Protein subunit vaccines contain fragments of protein, like the SARS-CoV-2 spike protein, that have a few advantages over other vaccine technologies. Primarily, they are easier and cheaper to produce compared to those containing whole pathogens and are also highly stable. Additionally, protein vaccines are considered safer than vaccines derived from live viruses, posing minimal risk of side effects.^50^ One challenge with protein-based vaccines is that they are less likely to be recognized by immune cells due to a lack of pathogen-associated molecular patterns within the antigen. To solve this problem, the codelivery of antigen and adjuvant is often required to increase recognition and the creation of T lymphocytes. Research has shown that MOF nanoparticles can serve as vehicles for the codelivery of antigens and adjuvants, improving the efficacy of protein-based vaccines.

Yang’s group found a reduction-responsive delivery system mimicking pathogenic vaccines using a safe and tunable MOF, MIL-101(Fe)-NH_2_.^49^ They conjugated the antigenic protein OVA to the surface of the MOF via disulfide bonds, and CpG was co-loaded into the MOF via adsorption. The disulfide bonds between the antigen and the MOF allowed for the release of OVA only when it is in the reductive environment of the cytosol of APCs, generating a strong T-cell response. During *in vitro* tests, it was found that utilizing MOFs for the delivery system allowed for the codelivery necessary to induce a potent immune response, as the MOF significantly improved uptake efficiency and facilitated the internalization of the antigen and adjuvant by the same cell. Compared to other OVA, CpG and MOF mixtures, the immunostimulatory response was further exemplified by the higher number of cytokines and memory T cells produced using the MOF-S-S-OVA@CpG. Notably, with the codelivery of antigen and adjuvant enabled by MOFs, there was evidence for increased amounts of CD8^+^ memory T cells, a vital part of intracellular viral responses.

Essentially, MOF-based codelivery systems can significantly improve immune system recognition when delivering a protein for antigenic therapy, thereby increasing the utility and effectiveness of protein-based vaccines. Extending these ideas to COVID-19 and other protein vaccines, MOFs could be potentially used to circumvent issues generally presented with protein-based vaccine systems, which should be further explored.

### 3.3 Nucleic Acid Protection and Delivery

In addition to protein vaccines, novel vaccine research, particularly for SARS-CoV-2, has also focused on nucleic acid vaccination technology. Both DNA- and mRNA-based vaccines have several advantages, such as their possible specificity to key proteins and the unneeded inclusion of immunodominant proteins that are dangerous or mostly irrelevant for protection. DNA vaccines are based on plasmid DNA (pDNA) that contain the DNA sequence of the antigens of interest to induce cellular and humoral immunological responses.^51^ For instance, DNA vaccines for the SARS-CoV-2 virus would contain the DNA sequence of the spike protein; once the vaccine is inoculated and sent into cells, it will be translated, and the spike protein will be released from the cell. The body should recognize the foreign protein and create antibodies against the virus. The advantages of DNA vaccines include their ease of production and efficacy in creating an immune response. However, there are still some concerns with DNA vaccines: DNA vaccines may trigger anti-DNA immune responses due to the use of prokaryotic DNA vectors.^52^

DNA vaccines do not require the rigorous cold-chain storage methods that other vaccines require, but their stability during storage and transportation is heavily dependent on the stabilization techniques used to keep them from degrading.^52^ MOFs can potentially improve DNA vaccines by serving as a nonviral vector to deliver DNA while protecting the encapsulated DNA from degradation.

MOFs can help deliver DNA through the formation of DNA@MOF biocomposites. Poddar et al. demonstrated the encapsulation of a complete gene set using ZIF-8 and a green fluorescent protein plasmid (plGFP) as a reporter.^53^ Mammalian cells were transfected with plGFP@ZIF-8 and examined for fluorescence to determine whether the plasmid had been transcribed and translated. Results indicated that the encapsulated gene retained functionality (with no DNA damage), consistent expression, and no signs of cytotoxicity. Although this study is not directly on DNA vaccines, it elucidated the viability of using MOFs as effective delivery systems for DNA within cells. The nonviral entry of DNA would address many safety concerns often associated with using viral vectors, including the potential to cause disease or over-replication of attenuated viruses.

MOFs can significantly improve the viability of DNA vaccines by providing protection. Li et al. used ZIF-8 for pDNA encapsulation and showed that the MOF could effectively protect the plasmid pEGFP-C1 against enzymatic degradation.^54^ Through the use of polyethylenimine (PEI), pEGFP-C1@ZIF-8-PEI exhibited increased loading of pDNA due to the enhanced electrostatic interactions between pDNA and PEI. These results indicate that ZIF-8 can effectively uptake and deliver pDNA at comparable or better rates than current methods. Since DNA vaccines also use pDNA for delivery, this approach could be extended to the intracellular delivery of such vaccines. Not only would Li’s methods potentially improve the efficacy of the MOFs used for vaccine protection, but they could also promote the mass production of DNA vaccines due to the economical synthesis. Although no DNA vaccines are currently approved for human use, using MOFs to encapsulate these vaccines could potentially expedite the approval and use of DNA vaccines.

Furthermore, there has been an increasing interest in mRNA (mRNA) vaccines and therapies due to their transient nature and the fact that mRNA can better bypass the barrier of the nuclear membrane compared to its DNA counterpart. During the pandemic, mRNA vaccines have gained increasing attention due to their “double immunity” inducing nature, which creates antigens for viruses and encourages killer cell production, generating a stronger immune response.^55^

The main obstacle to mRNA-based vaccines is the molecule’s susceptibility to enzymatic degradation. Previous work has shown that catiomers with elevated charge density and higher molecular weight would provide a higher degree of stability and protection for mRNA against enzymes like RNases.^56^ Sun’s group took these principles and synthesized a dendritic cationic Zr-based MOF known as MOF-poly(glycidyl methacrylate)-ethanolamine, or MOF-PGMA(EA), that was capable of effectively condensing the mRNA. The MOF-PGMA(EA) complex had higher colloidal stability than the PGMA(EA) complex alone, and more mRNA stayed intact in an RNase serum when complexed with MOF-PGMA(EA) as opposed to PGMA(EA).^57^ The MOF-mRNA complex also showed higher cellular uptake, possibly improving gene expression. These results indicate that MOFs can potentially increase the physiological deliverability of mRNA, which would help improve the function and efficacy of mRNA-based vaccines.

Although research on MOFs used for mRNA delivery is still limited, MOFs have been previously studied for the delivery of biomacromolecules with structural similarity to mRNA. Expanding some of these techniques to mRNA protection and delivery is possible. For instance, both ssDNA and mRNA have one strand, and ssDNA also retains other structural features common among nucleic acids. Peng et al. designed four isoreticular MOFs (Ni-IRMOF-74-II to -V) and tested their ability to transfect ssDNA.^41^ Each MOF in the series had different porosity, which was controlled by using organic linkers of varying lengths. These linkers contained salicylic acid to account for biocompatibility and were coordinated with Ni^2+^ through multiple oxygen atoms. The group tested the uptake, protection, and release of an ssDNA of 33 nucleotides in the MOFs. Fluorescent labels confirmed the uptake of ssDNA into the pores of the four MOFs. Afterward, a measure of protection was assessed by submerging the ssDNA-loaded MOFs into the fetal bovine serum to mimic physiological conditions. Remarkably, compared to the control with no protection, the Ni-IRMOF-74 series offered a 95% survival rate of the ssDNA. Ni-IRMOF-74-II was found to have the highest release rate (55%) of the ssDNA. Overall, the group determined that Ni-IRMOF-74-II and -III are the most effective for ssDNA transfection as they had the lowest strength of interactions between the ssDNA and the MOF pores, thus allowing them to not only hold the ssDNA inside the pore but also release it on demand. This MOF-stabilization method could be extended to mRNA, which is also prone to degradation in physiological fluid and the extracellular environment.

**Figure 2:**
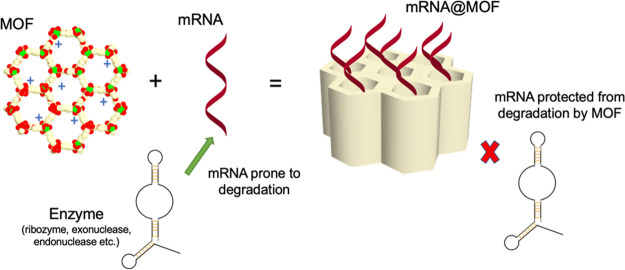
A single-stranded biomolecule, such as mRNA, can be incorporated into MOF pores for protection against degradation from ribozymes and other enzymes with cleaving activity.

Additionally, MOFs have been studied for the encapsulation and delivery of small interfering RNA (siRNA), which could also be extended to their potential applications in mRNA delivery. siRNA is a form of double-stranded RNA that codes for specific genes and can be synthetically created to target disease-associated genes. Teplensky et al. tested the efficacy of nNU-1000 in loading, protecting, and delivering siRNA.^58^ After loading the siRNA into the MOF pores, an enzyme protection gel assay confirmed that the siRNA contained in the MOF complex was not cleaved by the enzyme. On the other hand, the naked siRNA disappears on the gel, confirming that the MOF could protect the siRNA from enzymatic degradation. The group at first observed inconsistent efficacy due to potential endosomal entrapment. By incorporating species that can open up endosomes, more consistent levels of gene knockdown were observed. Notably, the additional protection provided to siRNA by the MOF could potentially be extended to *in vivo* protection of mRNA as a vaccine subunit because of their structural similarity (**Figure 2**).

In another recent study, Chen et al. synthesized selenium and ruthenium nanoparticles for the uptake and release of siRNA.^59^ Being more geared toward cancer therapy, the group looked at selenium for its strong antitumor activity and low toxicity as well as ruthenium for its antimetastatic effects. They utilized a MOF from the MIL family, MIL-101, which is known to have high porosity. Through the reduction of Na_2_SeO_3_ and RuCl_3_, Se@MIL-101 and Ru@MIL-101 were synthesized, which were then mixed with siRNA in deionized water, allowing the siRNA to bind to metal ions on the external surfaces of the MOFs. While naked siRNA was observed to be completely degraded, Se/Ru@MIL-101-siRNA still showed a siRNA band, indicating that the MOFs were able to protect siRNA from RNase degradation. Successful internalization of the siRNA was also observed via endocytosis pathways, and fluorescence markers showed the escape of siRNA from endo/lysosomal encasement, indicating efficient release capacity for Se/Ru@MIL-101. Similar to Teplensky’s methods, there is potential to extend the protective nature of MOFs to developing nucleic acid-based vaccines.

### 3.4 Designing Vaccine-Protective Systems

There is some nuance required to develop MOF-based systems to protect biomolecules. For instance, many proteins and their charges are sensitive to pH, temperature, and the general chemical environment. MOFs requiring more extreme syntheses (e.g., high temperature or very acidic/basic environments) may not be suitable for these proteins, as these conditions may denature the protein. In addition, it is important to consider interactions between the metal, linker, and proteins to determine whether or not encapsulation is feasible, as hydrogen bonds, electrostatic interactions, and hydrophobic interactions may promote or hinder the binding of the protein to the MOF building blocks. ZIFs may be particularly promising for protein systems: a recent paper showed that proteins with both positive and negative charges at physiological pH could be encapsulated in ZIFs.^60^ Negatively charged proteins can promote the formation of ZIFs nearly instantly, resulting in ZIF-protein composites. This is likely attributed to the electronic attraction of the negatively charged protein to the positively charged zinc ion of ZIFs. In contrast, positively charged proteins either took much longer time to form ZIFs or did not lead to the formation of the desired ZIFs. Many established biocompatible MOFs are constructed from positively charged metal ions, which may make encapsulating positively charged proteins more challenging. Future work might look at adding aspartate and glutamate to neutralize the charge of the protein without making significant alterations in their structure and function or functionalizing the surfaces of MOFs to improve the formation rates of the MOF-protein composites.

Although proteins can exist in various charged states depending on their sequences, nucleic acids are negatively charged polymers due to the highly negative phosphate backbone. This makes MOFs an even stronger candidate for designing nucleic-acid vaccines since the positively charged metal ions can stabilize the negatively charged polymers.

In summary, recent literature demonstrates that MOFs have great potential to function as nanoparticulate delivery systems for vaccine and macromolecule subunit protection. Not only do MOFs have the capability to prevent degradation, prerelease, and burst release of encapsulated cargos but also they can enhance intracellular uptake and create an environment that allows for antigen and adjuvant codelivery, significantly enhancing immune responses.

## 4 Antiviral Activity

**Figure 3:**
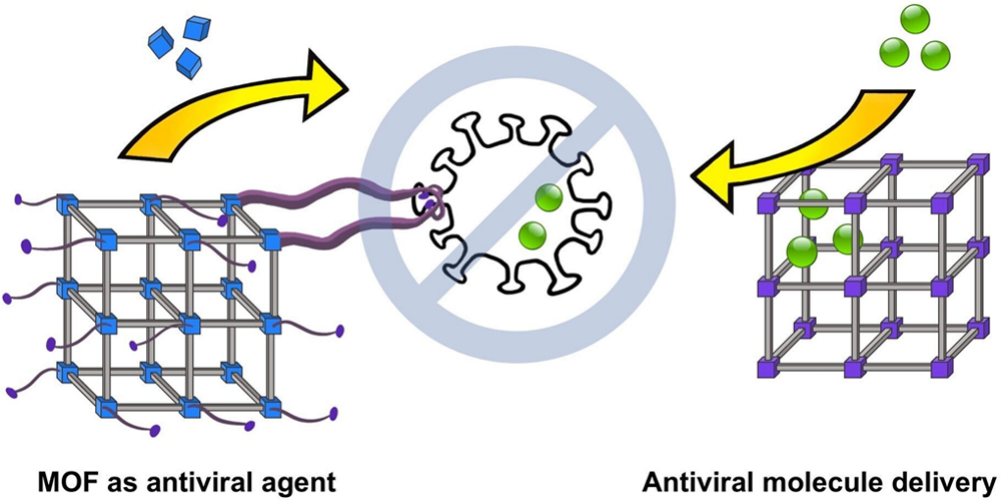
Mechanisms of antiviral activity using MOFs. MOFs can act as antiviral agents on their own (for example, through metal ion release or binding viral proteins), or they can be loaded with antiviral agents and functionalized to target viral cells.

Infectious diseases caused by viruses can pose widespread public health risks. The COVID-19 pandemic caused by SARS-CoV-2, flu caused by influenza viruses, and AIDS caused by human immunodeficiency virus (HIV) are just a few prominent examples. Unfortunately, the high genetic adaptability of viruses renders many current antiviral drugs less effective or ineffective over time, especially with increased drug usage.^61^ For example, influenza viruses change from year to year, or even within one flu season, which requires the flu vaccine composition to be updated annually based on which strains are predicted to circulate the most. Antiviral resistance raises the need to discover new compounds with antiviral activity and ways to enhance the clinical efficacy of existing antiviral drugs. Studies have shown that MOFs can be used to improve antiviral treatment by delivering antiviral drugs or directly inactivating viruses (**Figure 3**).^62−64^

### 4.1 Antiviral Drug Delivery using MOFs

One major mode of antiviral activity using MOFs is to encapsulate antiviral drugs into MOFs and deliver the drugs to the target cells. For example, Agostoni et al. loaded azidothymidine triphosphate (AZT-TP), a nucleoside reverse transcriptase inhibitor (NRTI), into MIL-100 nanoMOFs for delivery into major HIV target cells.^62^ When triphosphorylated, NRTIs are effective anti-HIV drugs because they inhibit the synthesis of proviral DNA. MIL-100 was chosen as a drug carrier because its Fe(III) clusters contribute to physiological stability and biocompatibility, and the MOF also has high porosity for drug encapsulation. AZT-TP was encapsulated through coordination with the Fe(III) clusters, quickly reaching a 24 wt % loading. The encapsulated AZT-TP was released upon exposure to the PBS buffer, as the free phosphates could compete for coordination with Fe(III). The MOF achieved a sustained release of AZT-TP into major HIV target cells, human peripheral blood mononuclear cells (PBMC), *in vitro*. Using MIL-100 as a drug carrier significantly increased the cellular uptake of AZT-TP, which overcame the poor stability of AZT-TP in biological media and its inability to cross the hydrophobic cell membrane as a hydrophilic molecule. Since MIL-100 was able to carry the active triphosphorylated form of the drug, this technique bypasses the need for intracellular kinases to triphosphorylate AZT, which has been the main barrier to the drug’s clinical efficiency.

### 4.2 MOFs and MOF Nanoparticles as Antiviral Agents

In addition to being used for antiviral drug delivery, some MOF nanoparticles themselves exhibit antiviral activity.^63^ Many metals, including copper, zinc, and silver, have shown some level of antiviral activity which makes MOFs based on these ions excellent candidates for neutralizing viral threats. For example, an antiviral MOF core–shell nanocomposite, Cu@ZIF-8 nanowires (NWs), was synthesized by growing a layer of ZIF-8 on the surface of pluronic acid-coated Cu NWs.^63^ ZIF-8 was used to coat the NWs to slow copper ion release and thus reduce the risk of copper-induced toxicity while maintaining antiviral activity. After VeroE6 kidney epithelial cells were infected with SARS-CoV-2 and incubated with Cu@ZIF-8 NWs *in vitro*, qRT-PCR was performed on viral RNA extracted from the supernatant. In the cells treated with 1 μg of Cu@ZIF-8 NWs, 37.6% and 54.6% virus replication inhibition were achieved for the envelope protein and nucleocapsid protein gene sequences, respectively. In contrast, the Cu NWs on their own led to 99% viral cell viability after 48 h. The superior antiviral activity of the MOF nanocomposite may be attributed to the presence of both the copper nanowires and zinc ions from the MOF.^63^ Copper seems to be an effective antiviral agent; another study found that SARS-CoV-2 was not viable on copper surfaces for over 4 h.^64^ In addition, 99% of host kidney epithelial cells remained viable after 48 h of exposure to Cu@ZIF-8 NWs, indicating negligible cytotoxicity, while the bare Cu NWs led to a significant decrease in host cell viability after exposure to a low dosage (50 μg/mL). It was also shown that cytotoxicity increases over time due to the sustained release of copper ions. Furthermore, the MOF allowed the Cu@ZIF-8 NWs to serve as excellent filters. Higher concentrations of the MOF composite showed filtration efficiencies of 70–80% for particles of 0.3 μm. As particle size increased above 1.0 μm, Cu@ZIF-8-NWs showed filtration efficiencies very similar to the N95 media that form the KN95 facemasks.

As mentioned earlier, many MOFs can be surface functionalized. The efficacy of MOF systems may be enhanced by functionalizing with other antiviral agents such as folic acid or carotenoids. One example is a proof-of-concept study by Desai et al., which showed that MOFs can be surface-functionalized with folic acid (FA), nystatin (Nys), or tenofovir (Teno) and bind to viral capsid proteins.^65^ This would immobilize the viruses and prevent them from replicating. These organic compounds were chosen because the terminal carboxylate groups in FA and Nys, and the phosphonate group in Teno increase the likelihood of binding viral capsid proteins. UiO-66-NO_2_, UiO-66-NO_2_-FA, and UiO-66-NO_2_-Teno effectively bound the SARS-CoV-2 spike protein in water (100%, 85%, and 71% binding, respectively). Moreover, the hydrophilic pores of these MOFs are expected to dehydrate virus-containing aerosols and thus inactivate the viruses. Not only can these MOFs be incorporated into fibers to develop antiviral PPE, but they can also be promising for biomedical or air purification purposes.

## 5 Antibacterial Activity

Bacterial infections are another type of infectious disease with similar modes of transmission as viral infections. Much like viruses, bacterial infections can range from mild to severe, depending on the strain of bacteria. The most famous instance of a widespread bacterial infection is the Bubonic plague, commonly referred to as the black death, which is infamous for its morbidity and mortality rate. Antibiotics have been an important and effective treatment for bacterial infections like those caused by *E. coli*, *S. aureus*, and *H. pylori*. The mechanism of action of antibiotics usually involves preventing the replication of bacteria or killing bacteria by interfering with their cellular functions and structures, such as by destroying cell walls. However, antibiotic overuse has caused many bacteria to gain multidrug resistance.^66,67^ Infections caused by antibiotic-resistant strains are increasingly challenging to treat, leading to longer hospital stays and higher medical costs.

**Figure 4:**
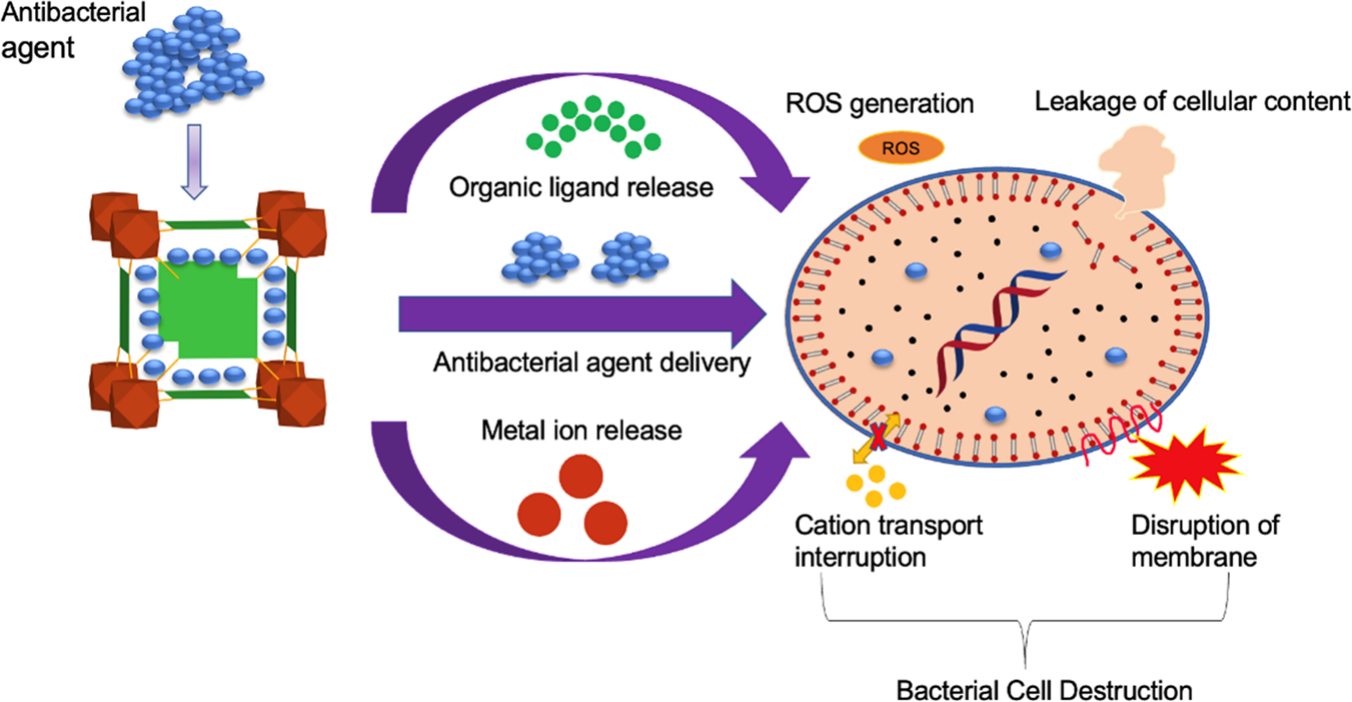
Common mechanisms and causes of antibacterial activity using MOFs (ROS: reactive oxygen species).

MOFs have been investigated as viable candidates for treating bacterial infections.^68−70^ The robust and tunable structures of MOFs allow for many different modes of action in treating bacterial infections. For example, MOFs can encapsulate antibiotics for enhanced cellular intake and provide alternative ways to bypass the problem of antibiotic resistance using their constituents. This section of the review highlights recent advances in using MOFs for antibacterial purposes based on the most explored mechanisms, including encapsulating and delivering antibacterial agents, photodynamic therapy (PDT), releasing metal ions or antibacterial linkers, or a combination of these mechanisms (**Figure 4**).^71^

### 5.1 Antibiotic Encapsulation and Delivery by MOFs

Encapsulation and delivery of antibiotics to target cells is one way that MOFs can be used to combat bacterial infections. For example, ZIF-8 was used to encapsulate ceftazidime, an important broad-spectrum antibiotic that can treat meningitis, *Salmonella* infection, and melioidosis, among other infections.^72^ In this study, ceftazidime was loaded into ZIF-8, and the resultant ceftazidime@ZIF-8 was tested for its effectiveness against*E. coli*. After ceftazidime@ZIF-8 was internalized into*E. coli* cells, the antibiotic was released at pH 5 (the environment of intracellular endosomes) upon degradation of ZIF-8. In addition to ceftazidime, the release of zinc ions from ZIF-8 penetrating the bacterial cell membrane also contributed to its antibacterial efficacy. Complete inhibition of*E. coli* growth was observed when 100 μg/mL ceftazidime@ZIF-8 was used. As bacteria can hide within macrophages, intracellular infections tend to be more challenging to treat. One advantage of ceftazidime@ZIF-8 is that it can release the antibiotic intracellularly, providing an effective strategy for treating intracellular infections. Intracellular antibiotic delivery has also been shown to cause enhanced antibacterial effects. Another advantage of this strategy is that fewer reinjections of ceftazidime were needed over the typical 10+ day acute treatment for melioidosis. It takes time for ceftazidime@ZIF-8 to gradually degrade and release the encapsulated ceftazidime; therefore, a desirable sustained release of the antibiotic can be achieved using MOFs.

### 5.2 Antibacterial Photodynamic Therapy Using MOFs

Another strategy for treating bacterial infections with MOFs is photodynamic therapy (PDT). PDT relies on the generation of reactive oxygen species (ROS) by a photosensitizer in drug-resistant bacteria. ROS can kill bacteria by placing high oxidative stress on their metabolic pathways.^73^ Traditional photosensitizers can suffer from aggregation and reduced efficiency under physiological conditions. However, the covalently incorporated or encapsulated photosensitizing moieties in MOFs are spatially isolated, minimizing their aggregation to achieve high efficiency.

Bagchi et al. investigated the incorporation of a hydrophobic photosensitizer squaraine into ZIF-8 for antibacterial PDT.^74^ Squaraine was postsynthetically attached to ZIF-8, and the resulting material, ZIF8-SQ, preserved the photoactivity of squaraine while preventing the self-aggregation of hydrophobic squaraine. The antibacterial effects of ZIF8-SQ were demonstrated by incubating methicillin-resistant*Staphylococcus aureus* (MRSA) with various concentrations of ZIF8-SQ, with and without red light irradiations. It was found that ZIF8-SQ exhibited highly efficient antibacterial activity due to the generation of cytotoxic ROS under red light. The orientation of the squaraine molecules within the MOF allowed for electrons to stay in the excited state for longer periods of time, promoting ROS generation and effectively increasing antibacterial activity.

In addition to attaching and encapsulating photosensitizers onto/into MOFs, photoactive linkers can be directly incorporated into MOFs as their building blocks.^75^ In particular, MOFs with porphyrin linkers have been extensively studied for PDT.^76−79^ For example, Chen et al. modified a MOF with porphyrin linkers, PCN-224(Zr), by incorporating Ti into the Zr nodes to obtain PCN-224(Zr/Ti) while preserving the MOF structure.^80^ The Ti incorporation increased the ROS generation of the MOF, which was found highly effective against several strains of bacteria, including multidrug-resistant*E. coli*,*A baumannii*,*A. aureus*, and*S. epidermidis*. The biocompatibility of PCN-224(Zr/Ti) was also tested *in vivo* via intravenous injection into rats and followed by H&E stains of the major organs 14 days later, which showed the MOF had negligible toxicity. This work demonstrated that efficient and biocompatible MOF photosensitizers are promising materials for PDT in treating bacterial infections.

### 5.3 Antimicrobial Activity of MOFs

There are numerous mechanisms by which MOFs have shown antimicrobial activity. Many bacteria are sensitive to the presence of metal ions. MOFs essentially act as a reservoir of such metal ions. Gram-positive and Gram-negative bacteria are both negatively charged, so there are strong electrostatic interactions between the cell membranes and the unsaturated metal sites of MOFs or metal ions released from the gradual degradation of MOFs, causing cell lysis. Ag^+^, Al^3+^, Cu^2+^, and Zn^2+^ have all been shown to disrupt metabolic activity in bacteria, making them excellent choices as metal ions when designing bactericidal MOFs.^81^ In addition, Co^2+^ has also demonstrated antibacterial activity.^82^ However, more caution needs to be taken when incorporating Co^2+^ into materials that may come into contact with humans due to the potential of cobalt toxicity.^83^

In a MOF, both the metal ions and the organic linkers can serve as antimicrobials. For instance, taking advantage of the well-documented antimicrobial activity of Ag^+^, Jaffrès et al. created a silver-based MOF with 3-phosphonobenzoate as a linker that showed high potency against several bacterial strains, including*S. aureus* and*E. coli*.^84^ While antibiotics like kanamycin and ampicillin were not active against all the strains tested by the Jaffrès group, the MOF showed consistent antimicrobial activity. This study demonstrated that the nonspecific nature of MOFs endows them with broad antimicrobial activity. The organic linkers in MOFs can also impart antimicrobial activity to these structures. In addition to generating ROS as discussed above, linkers like azelaic acid, a well-documented broad-spectrum antibiotic often used to combat acne, can be incorporated into MOFs to inactivate various types of bacteria, including*S. aureus*, *E. coli*, and*Pseudomonas aeruginosa*.^85,86^

In addition to directly incorporating building blocks with antimicrobial properties in MOF design, nanoparticles with antimicrobial activities can be introduced into MOFs through encapsulation or postsynthetic modifications. Tian et al. recently reported ZIF-8 doped with Ag(I) nanoparticles coated on a stainless-steel mesh. The Ag(I)-doped ZIF-8 exhibited strong antimicrobial activity, which showed only 7% bacterial growth after incubation with *E. coli*, while the control stainless-steel mesh showed an 80 ± 6% growth.^87^ Although the initial study was for applications in water cleansing, such MOF composites may have potential applications as antibacterial medicine. Many zinc MOFs, like ZIF-8, are biocompatible and can serve as a vessel to deliver bactericidal agents, such as the Ag(I) nanoparticles.

## 6 Personal Protective Equipment

The COVID-19 pandemic led to a renewed interest in improving currently available forms of PPE. With a continued need for PPE in various industries, there has been a recent push to advance currently available protective technologies. Recently, MOFs have been employed to produce filters, masks, and other materials with biocidal activity for public health protection. MOFs are great candidates for textile materials because of their vast design possibilities; their easily manipulated structure and porosity also present the opportunity for high filtration efficiency and pathogenic protection.^65,88^

### 6.1 Filters

Fibrous filters and meshwork are the most commonly employed barriers to keep particulate matter out; however, these filters have limitations in blocking microorganisms like bacteria and viruses and can quickly become a pathogenic breeding ground. Li et al. explored the use of MOFs to construct an integrated material that not only removed particulate matter but also killed off germs entirely.^89^ The porous nature and tunable structure of MOFs enable them to possess a multifunction for filtration and ROS generation, capable of removing particulate matter, oxidizing pollutants, and destroying bacteria. Li et al. studied five MOFs and found that ZIF-8 on fabric exhibited the best photocatalytic efficiency, outperforming previously studied semiconductors. After 30 min in an enclosed air environment containing aerosols of *E. coli* suspension, ZIF-8 showed practically 100% inactivation of *E. coli*. Compared to <89%*E. coli* inactivation using the control fabric, this result indicates that bactericidal MOFs can be used as effective air filters to create sterile environments and prevent the spread of infectious diseases. Bactericidal MOFs have also been used to develop self-cleaning membranes that filter pollutants in solution to obtain clean water and prevent biofouling.^90,91^

In other studies, MOFs have also been investigated as air filters that remove toxic gases, volatile organic compounds, and/or particulate matter.^92−94^ These MOF textiles can be synthesized using solvent-free hot pressing and electrophoretic deposition techniques. MOF textiles with antiviral/antibacterial properties may be useful for designing air filters/purifiers that specifically combat airborne pathogens.

### 6.2 Masks and Wearable PPE

Because face masks are one of the main ways to prevent the spread of viral infections, treating face masks with a microbicidal material would provide higher efficacy against infections. Interestingly, Li’s group employed the ZIF-8 filter they developed (discussed under section 6.1), also known as a MOFilter, to design a trilayer mask.^89^ The study showed that the bottom layer of the mask (which, in practice, would be in direct contact with human skin) had no measurable amount of bacteria half an hour after the mask was exposed to *E. coli* suspensions.

In addition to relying on the ROS generated from photoactive MOFs, self-sanitizing face masks utilizing other microbicidal agents, such as metal ions, were also reported.^7,63^ For example, antibacterial face masks were fabricated by functionalizing Cu@ZIF-8 NWs onto three-layer filter media made of melt-blown polypropylene, a hydrophobic polymer.^63^ One concern was whether Cu@ZIF-8 NWs would undesirably shed from the filter during filtration and potentially enter the human throat or oral cavity. To test this, a condensation particle counter was used, and a particle count of zero indicated a negligible loss of the nanowires from the filter. At concentrations of Cu@ZIF-8 NWs above 0.25 mg/mL, the filtration efficiency of the filter media was higher than that of untreated filter media. This higher efficiency was attributed to the simultaneous and sustained release of copper and zinc ions on the functionalized filters, which would inactivate any microbes breathed out by someone wearing a face mask functionalized with Cu@ZIF-8 NWs. The reusability of this filter material in face masks was also proposed due to this self-sanitizing effect. Future studies will test the antiviral activity of the functionalized mask when exposed to virus-containing aerosols, which is important because most respiratory disease viruses can be spread through respiratory droplets and aerosol particles.

In addition, Cheung et al. recently developed an amine-functionalized Zr-based MOF, UiO-66-NH_2_, as biocidal textiles.^7^ The amine groups served as a carrier for the active chlorine molecules that acted as the biocidal agent. The MOF was first coated onto a fiber, polyethylene terephthalate, which was then chlorinated by immersion into a commercial bleaching agent to form the activated chlorine-loaded MOF/fiber composite with the amine linker binding to the *N*-chlorine biocide. The composite was tested by exposing it to the SARS-CoV-2 virus. Compared to the fiber on its own as well as a nonchlorinated MOF/fiber composite, the chlorinated MOF/fiber composite led to a significant delay in viral growth within the first two days of measurement. Notably, prominent decreases in bacterial activity were observed for the MOF/fiber composite loaded with just 0.18% activated chlorine. The MOF layer also displayed excellent stability and regenerability, which is an exciting feature of these MOF textiles as they can form reusable PPE to minimize medical waste. The effectiveness and reusability of the MOF textile composite demonstrated the potential of using MOFs to develop inexpensive, antiviral PPE with biocidal properties.

**Figure 5:**
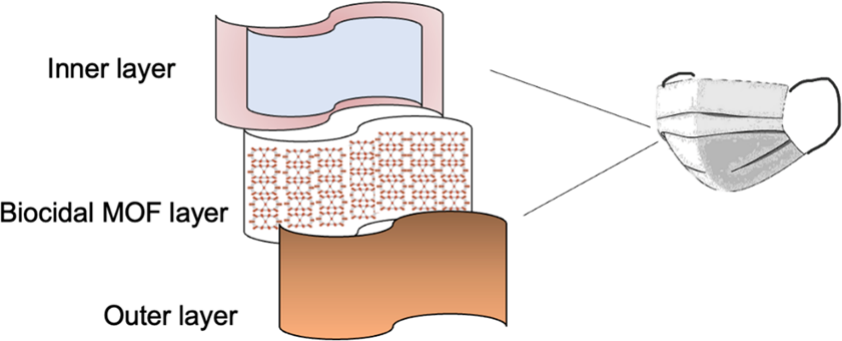
A proposed sample multilayer biocidal facemask with outer and inner layers made of nonwoven fabrics (similar to other masks^64^), a biocidal and photocatalytic MOF layer, and an antioxidant film (shown in light blue) that could effectively manage any escaped reactive oxygen species.

There are numerous benefits of MOF materials like the MOFilter or self-cleaning textiles, especially since these materials can kill bacteria at rates comparable to antibiotics without the issue of drug resistance. However, there is a major question about whether the ROS generated from these materials compromises their safety. Several papers previously mentioned that the ROS generated by the MOF stayed within the porous structure of the MOF.^78^ However, careful examinations must be conducted for each MOFilter to ensure that ROS are not internalized by the users, especially if the materials become part of face coverings. Among other problems, ROS can cause DNA strands to break, which could eventually lead to diseases like cancer.^95^ Thus, before these materials are extended to mass production, it must be confirmed that the generated ROS will not impact PPE users. One possible solution could be developing PPE with MOF and antioxidant layers (**Figure 5**). It was recently reported that antioxidant polymers could be crafted into a film;^96^ when combined with photoactive MOF, these biomaterials could potentially form highly effective and safe PPE.

## 7 Conclusions

This review summarized recent efforts in using MOFs to construct practical defense systems against infectious diseases. We highlighted several approaches for vaccine protection and studies that could be extended to developing modern-day vaccine technologies. In addition, we discussed various ways MOFs can be used for antiviral/antibacterial treatments and specific diseases for which these treatments can be effective. Lastly, we reviewed various MOFs utilized in creating modern, facile, and effective PPE against infectious diseases. The intersection of coordination chemistry and clinical chemistry has led to many exciting discoveries to improve defense against infectious diseases. Considering MOFs’ promising capability in assisting or serving as defense systems, future work would include further exploration of MOF protection for mRNA and DNA, developing facile syntheses for mRNA- and DNA-based vaccines, and testing their efficacy.

The COVID-19 pandemic is the most recent example of the ongoing need for vigorously effective defense systems that can stop the spread and inactivate infectious agents once they have entered the body. Most MOFs currently being studied for preventing and treating infectious diseases are still at the preclinical stage. There is much room for future studies on how we can utilize MOFs safely and effectively to create novel vaccines, develop new therapies, and improve intracellular delivery. Furthermore, MOFs will likely be implemented in next-generation antibacterial and antiviral PPE, which can help stall the spread of infectious diseases and prevent future pandemics.

## Supporting Information Available

Names, metal ions, and ligands of MOFs and their applications related to the defense and treatment of infectious diseases (PDF)
