# Supplementary material for: Constructing Physiological Defense Systems against Infectious Disease with Metal–Organic Frameworks: A Review
Source: ACS Appl Bio Mater. 2023 Aug 10;6(8):3052–65. doi: 10.1021/acsabm.3c00391 (PMC10445270; doi:10.1021/acsabm.3c00391)
Supplement: Supplementary file 1 — mt3c00391_si_001.pdf [file mt3c00391_si_001.pdf]

Supporting Information

Constructing physiological defense systems against infectious disease with metal-organic frameworks: a review

Nikita O. Mishra,[a,†] Alisa S. Quon,[a] Anna Nguyen,[a,‡] Edgar K. Papazyan, [a,‡] Yajiao Hao,[a] Yangyang Liu[a,*]

[a] Department of Chemistry and Biochemistry, California State University, Los Angeles, 5151 State University Drive, Los Angeles, CA 90032, United States.

† Department of Bioengineering, Stanford University, Stanford, California, 94305, United States.

‡ California University of Science & Medicine, Colton, California, 92324, United States.

* Correspondence should be addressed to Y. L. (yliu114@calstatela.edu)

KEYWORDS: metal-organic frameworks, infectious disease, vaccines, macromolecule protection, antiviral, antibacterial, drug delivery, personal protective equipment

Table S1. The names, metal ions, and ligands of MOFs and their applications related to the defense and treatment of infectious diseases reported to date

MOF/MOF composite	Metal Ion	Ligand	Function	Ref.
ZIF-8	Zn^{2+}	2-Methylimidazole	Macromolecule and vaccine protection, filters/PPE, antibacterial	1-14
NU-1000	Zr^{4+}	1,3,6,8-tetrakis(p-benzoate)pyrene	Nucleic acid protection and delivery	13,15
MIL-100	Fe^{3+}	Benzene-1,3,5-tricarboxylate	Nucleic acid protection and delivery, antiviral drug delivery, antibacterial	13,16-19
MIL-53(Al)	Al^{3+}	Terephthalic acid	Filters/PPE	11
MFM-300(In)	In^{3+}	Biphenyl-3,3′,5,5′-tetracarboxylic acid	Filters/PPE	11
UiO-66	Zr^{4+}	Terephthalic acid	Filters/PPE, virucidal against HIV-1, antibacterial	11,20, 21
Ag(I) BioMOF	Ag^{+}	1,3,5-triaza-7-phosphaadamantane and pyromellitic acid	Virucidal against HAdV-36, antibacterial	22
HKUST-1	Cu^{2+}	Benzene-1,3,5-tricarboxylate	Virucidal against MS2 bacteriophage, antiviral drug delivery (hydroxychloroquinone), antibacterial	23,24
Thio@MIL-125-NH_2@CMC	Ti^{4+}	2-amino terephthalic acid	Virucidal against HSV1 and COX B4, antibacterial	25
MIL-101	Fe^{3+}	Aminoterephthalate	Antiviral drug delivery, antibacterial (amoxicillin, potassium clavulanate	13,17, 26-28

			loading)	
ZTIFs	Zn^{2+}	5,6-dimethyl benzimidazole, tetrazole	Antiviral drug encapsulation (oseltamivir, ritonavir)	29
$[AgL]_n$·nH2O (p-MOF)	Ag^+	4-Cyanobenzoate	Antibacterial (metal ion release)	30
Ag2(μ_2-PTA)(μ_3-PTA)(μ2-pga)(H2O)$]_n$·6H_2O	Ag^+	1,3,5-triaza-7-phosphaadamantane; 3-phenylglutaric acid	Antibacterial (metal ion release)	31
[Ag_2(μ_2-PTA)(μ_3-PTA)(Hpmal)$_2]_n$·2H_2O	Ag^+	1,3,5-triaza-7-phosphaadamantane; phenylmalonic acid	Antibacterial (metal ion release)	31
[Ag(μ_3-PTA)(Hdmga)$]_n$	Ag^+	1,3,5-triaza-7-phosphaadamantane; 3,3-dimethylglutaric acid	Antibacterial (metal ion release)	31
[Ag_2(μ-PTA)$_2$(μ-suc)$]_n$·2nH2O	Ag^+	1,3,5-triaza-7-phosphaadamantane; succinic acid	Antibacterial (metal ion release)	32
[Ag_2(μ-PTA)2(μ_4-adip)$]_n$·2nH_2O	Ag^+	1,3,5-triaza-7-phosphaadamantane; adipic acid	Antibacterial (metal ion release)	32
[Ag2(μ_4-PTA)(μ_4-mal)]n	Ag^+	1,3,5-triaza-7-phosphaadamantane; malonic acid	Antibacterial (metal ion release)	32
[Ag(NO_3)(μ_3-PTA = O)$]_n$	Ag^+	1,3,5-triaza-7-phosphaadamantane-7-oxide	Antibacterial (metal ion release)	33
[Ag_2(μ_2-SO_4)(μ_5-PTA = O)(H_2O)$]_n$	Ag^+	1,3,5-triaza-7-phosphaadamantane-7-oxide	Antibacterial (metal ion release)	33
[Ag(μ_3-PTA = S)$]_n$(NO_3)$_n$·nH2O	Ag^+	1,3,5-triaza-7-phosphaadamantane-7-sulfide	Antibacterial (metal ion release)	34

$[Ag_4(\mu_4\text{-PTA = S})(\mu_5\text{-PTA = S})(\mu_2\text{-}SO_4)_2(H_2O)_2]_n\cdot 2nH_2O$	Ag^+	1,3,5-triaza-7-phosphaadamantane-7-sulfide	Antibacterial (metal ion release)	34
$[Ag_2(O\text{-}IPA)(H_2O)\cdot(H_3O)]$	Ag^+	5-hydroxyisophthalic acid	Antibacterial (metal ion release)	35
$[Ag_5(PYDC)_2(OH)]$	Ag^+	pyridine-3, 5-dicarboxylic acid	Antibacterial (metal ion release)	35
AgMOF–CQDs	Ag^+	1,3,5-benzenetricarboxylic acid	Antibacterial (metal ion release)	36
GO–Ag-MOF	Ag^+	1,3,5-benzenetricarboxylic acid	Antibacterial (metal ion release)	37,38
Ag-MOF/polyamide thin-film	Ag^+	2-aminoterephthalic acid	Antibacterial (metal ion release)	39
Ag-MOF-polyamide thin membrane	Ag^+	2-aminoterephthalic acid	Antibacterial (metal ion release)	40
Ag-MOF/polyamide thin-film	Ag^+	2-aminoterephthalic acid	Antibacterial (metal ion release)	41
Ag-MOFs@CF	Ag^+	2-aminoterephthalic acid	Antibacterial (metal ion release)	42
TFC-BPA-Ag-MOF membrane	Ag^+	2-methylimidazole	Antibacterial (metal ion release)	43
Ag@MOF/CSNP	Ag^+	Pyridine-3,5-dicarboxylic acid	Antibacterial (metal ion release)	44
$Zn_2(oba)_2bpy$	Zn^{2+}	4,4-oxybisbenzoic acid; 4,4-bipyridine	Antibacterial (metal ion release)	45
Zn-SIM1	Zn^{2+}	4-Methyl-5-imidazolecarboxaldehyde	Antibacterial (metal ion release)	46
ZnBDC-Ctn	Zn^{2+}	1,4-benzenedicarboxylic acid	Antibacterial (metal ion release)	47

MOF-5	Zn^{2+}	1,4-benzenedicarboxylic acid	Antibacterial (metal ion release; ampicillin, kanamycin, tetracycline loading)	48-51
Zn-MOF	Zn^{2+}	1,4-benzenedicarboxylic acid	Antibacterial (metal ion release)	48
TMU-3	Zn^{2+}	1,4-benzenedicarboxylic acid	Antibacterial (metal ion release)	48
[ZnCu(bdc)$_2$(dabco)]	Zn^{2+}, Cu^{2+}	1,4-benzenedicarboxylic acid; 1,4-diazabicyclo [2.2.2]octane	Antibacterial (metal ion release)	52
M–Cu&Zn-MOFs	Zn^{2+}, Cu^{2+}	Niacin	Antibacterial (metal ion release)	53
Cu-SURMOF 2	Cu^{2+}	1,4-benzenedicarboxylic acid	Antibacterial (metal ion release)	54
Cu/H$_3$BTC	Cu^{2+}	1,3,5-benzenetricarboxylic acid	Antibacterial (metal ion release)	55
Cu-BTC/fibers	Cu^{2+}	1,3,5-benzenetricarboxylic acid	Antibacterial (metal ion release)	56,57
L-Glu-Cu	Cu^{2+}	*L*-Glutamic acid	Antibacterial (metal ion release)	58
[Cu$_2$(bdc)$_2$(dabco)]	Cu^{2+}	1,4-benzenedicarboxylic acid; 1,4-diazabicyclo [2.2.2]octane	Antibacterial (metal ion release)	59
Cu-MOF-74/PDA-PVDF membranes	Cu^{2+}	2,5-Dihydroxyterphthalic acid	Antibacterial (metal ion release)	60
Fe_3O_4@Cu-MOF	Cu^{2+}	2,6-Pyridine	Antibacterial (metal ion	61

		dicarboxylic acid	release)	
Cu-MOF-1/PLA fibrous membrane	Cu^{2+}	Citric acid	Antibacterial (metal ion release)	62
ZIF-67	Co^{2+}	2-methylimidazole	Antibacterial (metal ion release)	63-65
Co-SIM-1	Co^{2+}	4-Methyl-5-imidazolecarboxaldehyde	Antibacterial (metal ion release)	46,65, 66
L-Glu-Co	Co^{2+}	*L*-Glutamic acid	Antibacterial (metal ion release)	58
[Co(BDC)(Phen)H_2O]	Co^{2+}	1,4-benzenedicarboxylic acid; 1,10-phenanthroline	Antibacterial (metal ion release)	67
[Co(BDC)(DABCO)]	Co^{2+}	1,4-benzenedicarboxylic acid; 1,4-diazabicyclo [2.2.2]octane	Antibacterial (metal ion release)	67
Ni-MOFs	Ni^{2+}	2-methylimidazole	Antibacterial (metal ion release)	68
Cotton@silicate@ZIF(Ni)	Ni^{2+}	2-methylimidazole	Antibacterial (metal ion release)	63
L-Glu-Ni	Ni^{2+}	*L*-Glutamic acid	Antibacterial (metal ion release)	58
H-TiO_{2-x}@Ga-MOF	Ga^{3+}	Carbenicillin	Antibacterial (metal ion release)	69
$Ag_{2n}(BTEC)_{n/2}$ film	Ag^{+}	1,2,4,5-benzenetetracarboxylic acid	Antibacterial (metal active site)	70
Co-TDM	Co^{2+}	Tetrakis[(3,5-dicarboxyphenyl)-oxamethyl] methane acid	Antibacterial (metal active site)	71
AU-1	Ce^{4+}	4,4',4''-	Antibacterial (metal	72

		Nitrilotribenzoic acid	active site)	
$[Cu_2(Glu)_2(\mu\text{-}bpy)]\cdot x(H_2O)$	Cu^{2+}	Glutarate; 4,4’-bipyridine	Antibacterial (metal active site)	73
$[Cu_2(Glu)_2(\mu\text{-}bpa)]\cdot x(H_2O)$	Cu^{2+}	Glutarate; 1,2-bis(4-pyridyl) ethane	Antibacterial (metal active site)	73
$[Cu_2(Glu)_2(\mu\text{-}bpe)]\cdot x(H_2O)$	Cu^{2+}	Glutarate; 1,2-bis(4-pyridyl) ethylene	Antibacterial (metal active site)	73
$[Cu_2(Glu)_2(\mu\text{-}bpp)]\cdot x(H_2O)$	Cu^{2+}	Glutarate; 1,2-bis(4-pyridyl) propane	Antibacterial (metal active site)	73
AFP-Cu(II)	Cu^{2+}	5,5’-(Piperazine-1,4-diylbis(methylene)) bis(2-aminobenzoic acid)	Antibacterial (metal active site)	74
BioMIL-5	Zn^{2+}	Azelaic acid	Antibacterial (active ligand)	75
$[K_2(H_2AZE)(AZE)]$	K^{+}	Azelaic acid	Antibacterial (active ligand)	76
$H\text{-}TiO_{2-x}$@GaMOF	Ga^{3+}	Carbenicillin	Antibacterial (active ligand)	69
IRMOF-3	Zn^{2+}	2-aminoterephthalic acid	Antibacterial (active ligand, ampicillin and kanamycin loading)	50,77
UiO-67-bpydc-Ag	Zr^{2+}	2,2’-bipyridine-5,5’-dicarboxylic acid-Ag	Antibacterial (active ligand)	78
UiO-66-2COOAg	Zn^{2+}	Silver 1,2,4,5-benzenetetracarboxylate	Antibacterial (active ligand)	78
MIL-88B(Fe)	Fe^{3+}	2-aminoterephthalic acid	Antibacterial (active ligand, antimicrobial-	78,79

			loaded)	
Zn-BTC	Zn^{2+}	1,3,5-benzenetricarboxylic acid	Antibacterial (ampicillin and kanamycin loading)	50
CD-MOF	K^{+}	γ-cyclodextrin	Antibacterial (enrofloxacin, florfenicol, sulfadiazine loading)	80-82
$Zn_2(bdc)_2(dabco)$	Zn^{2+}	1,4-benzenedicarboxylic acid, 1,4-diazabicyclo [2.2.2] octane	Antibacterial (gentamicin and nalidixic acid loading)	83,84
Ni-CPO-27	Ni^{2+}	2,5-Dihydroxyterephthalate	Antibacterial (metronidazole loading, NO release)	85,86
Ni/Co-MOF@CMC	Ni^{2+}, Co^{2+}	1,4-benzenedicarboxylic acid	Antibacterial (tetracycline loading)	87
MOF-53	Fe^{3+}	1,4-benzenedicarboxylic acid	Antibacterial (vancomycin loading)	88,89
Ag@PVDF/PVP/Mg $(C_{10}H_{16}O_4)_2(H_2O)_2$	Mg^{2+}	Sebacic acid	Antibacterial (metal ion release)	90
$[Zn_4O(dmcapz)_3]$	Zn^{2+}	3,5-Dimethyl-4-carboxypyrazolato (dmcapz)	Antibacterial (antimicrobial-loaded)	91
RPM6-Zn	Zn^{2+}	Biphenyl-4,40 -dicarboxylate, 4,4’-azobispyridine	Antibacterial (antimicrobial-loaded)	92,93
Fe(III)-HMOF-5	Zn^{2+}, Fe^{3+}	1,4-benzenedicarboxylic acid	Antibacterial (antimicrobial-loaded)	94
Zn@MOF	Zn^{2+}	2-aminoterephthalic acid	Antibacterial (antimicrobial-loaded)	95

References

1. Li, S.; Dharmarwardana, M.; Welch, R. P.; Benjamin, C. E.; Shamir, A. M.; Nielsen, S. O.; Gassensmith, J. J. Investigation of Controlled Growth of Metal–Organic Frameworks on Anisotropic Virus Particles. *ACS Appl. Mater. Interfaces* **2018**, *10*, 18161-18169.

2. Zuo, Q.; Li, T.; Huang, L.; Liu, Z.; Xue, W. Macro-microporous ZIF-8 MOF complexed with lysosomal pH-adjusting hexadecylsulfonylfluoride as tumor vaccine delivery systems for improving anti-tumor cellular immunity. *Biomater. Sci.* **2023**, *11*, 5025-5045.

3. Li, S.; Dharmarwardana, M.; Welch, R. P.; Ren, Y.; Thompson, C. M.; Smaldone, R. A.; Gassensmith, J. J. Template-Directed Synthesis of Porous and Protective Core-Shell Bionanoparticles. *Angew. Chem., Int. Ed.* **2016**, *55*, 10691-10696.

4. Singh, R.; White, J. F.; de Vries, M.; Beddome, G.; Dai, M.; Bean, A. G.; Mulet, X.; Layton, D.; Doherty, C. M. Biomimetic metal-organic frameworks as protective scaffolds for live-virus encapsulation and vaccine stabilization. *Acta Biomater.* **2022**, *142*, 320-331.

5. Luzuriaga, M. A.; Welch, R. P.; Dharmarwardana, M.; Benjamin, C. E.; Li, S.; Shahrivarkevishahi, A.; Popal, S.; Tuong, L. H.; Creswell, C. T.; Gassensmith, J. J. Enhanced Stability and Controlled Delivery of MOF-Encapsulated Vaccines and Their Immunogenic Response In Vivo. *ACS Appl. Mater. Interfaces* **2019**, *11*, 9740-9746.

6. Li, Y.; Zhang, K.; Liu, P.; Chen, M.; Zhong, Y.; Ye, Q.; Wei, M. Q.; Zhao, H.; Tang, Z. Encapsulation of Plasmid DNA by Nanoscale Metal–Organic Frameworks for Efficient Gene Transportation and Expression. *Adv. Mater. (Weinheim, Ger.)* **2019**, *31*, e1901570-n/a.

7. Poddar, A.; Conesa, J. J.; Liang, K.; Dhakal, S.; Reineck, P.; Bryant, G.; Pereiro, E.; Ricco, R.; Amenitsch, H.; Doonan, C.; Mulet, X.; Doherty, C. M.; Falcaro, P.; Shukla, R. Encapsulation, Visualization and Expression of Genes with Biomimetically Mineralized Zeolitic Imidazolate Framework-8 (ZIF-8). *Small* **2019**, *15*(36).

8. Nong, W.; Wu, J.; Ghiladi, R. A.; Guan, Y. The structural appeal of metal–organic frameworks in antimicrobial applications. *Coord. Chem. Rev.* **2021**, *442*, 214007.

9. Li, Y.; Liu, L.; Meng, T.; Wang, L.; Xie, Z. Structural Engineering of Ionic MOF@COF Heterointerface for Exciton-Boosting Sunlight-Driven Photocatalytic Filter. *ACS Nano* **2023**, *17*(3), 2932, .

10. Li, X.; Zhu, G.; Tang, M.; Li, T.; Wang, C.; Song, X.; Zhang, S.; Zhu, J.; He, X.; Hakkarainen, M.; Xu, H. Biodegradable MOFilters for Effective Air Filtration and Sterilization by Coupling MOF Functionalization and Mechanical Polarization of Fibrous Poly(lactic acid). *ACS Appl. Mater. Interfaces* **2023**, *15*(22), 26812, .

11. Zhang, K.; Huo, Q.; Zhou, Y.; Wang, H.; Li, G.; Wang, Y.; Wang, Y. Textiles/Metal–Organic Frameworks Composites as Flexible Air Filters for Efficient Particulate Matter Removal. *ACS Appl. Mater. Interfaces* **2019**, *11*, 17368-17374.

12. Bian, Y.; Niu, Z.; Wang, S.; Pan, Y.; Zhang, L.; Chen, C. Removal of Size-Dependent Submicron Particles Using Metal–Organic Framework-Based Nanofiber Air Filters. *ACS Appl. Mater. Interfaces* **2022**, *14*(20), 23570, .

13. He, Y.; Li, D.; Wu, L.; Yin, X.; Zhang, X.; Patterson, L. H.; Zhang, J. Metal-Organic Frameworks for Gene Therapy and Detection. *Adv. Funct. Mater.* **2023**, *33*, 2212277-n/a.

14. Wang, Y.; Shahi, P. K.; Xie, R.; Zhang, H.; Abdeen, A. A.; Yodsanit, N.; Ma, Z.; Saha, K.; Pattnaik, B. R.; Gong, S. A pH-responsive silica–metal–organic framework hybrid nanoparticle for the delivery of hydrophilic drugs, nucleic acids, and CRISPR-Cas9 genome-editing machineries. *J. Controlled Release* **2020**, *324*, 194-203.

15. Teplensky, M. H.; Fantham, M.; Poudel, C.; Hockings, C.; Lu, M.; Guna, A.; Aragones-Anglada, M.; Moghadam, P. Z.; Li, P.; Farha, O. K.; Bernaldo de Quirós Fernández, S.; Richards, F. M.; Jodrell, D. I.; Kaminski Schierle, G.; Kaminski, C. F.; Fairen-Jimenez, D. A Highly Porous Metal-Organic Framework System to Deliver Payloads for Gene Knockdown. *Chem* **2019**, *5*, 2926-2941.

16. Hidalgo, T.; Alonso-Nocelo, M.; Bouzo, B. L.; Reimondez-Troitiño, S.; Abuin-Redondo, C.; de la Fuente, M.; Horcajada, P. Biocompatible iron(iii) carboxylate metal–organic frameworks as promising RNA nanocarriers. *Nanoscale* **2020**, *12*, 4839-4845.

17. Clayette, P.; Heurtaux, D.; Baati, T.; Kreuz, C.; Sebrie, C.; Bories, P.; Chalati, T.; Horcajada, P.; Gillet, B.; Serre, C.; Marsaud, V.; Hwang, Y. K.; Couvreur, P.; Gref, R.; Eubank, J. F.; Chang, J.; Cynober, L.; Férey, G.; Gil, S. Porous metal-organic-framework nanoscale carriers as a potential platform for drug delivery and imaging. *Nat. Mater.* **2010**, *9*, 172-178.

18. Duan, C.; Liu, C.; Meng, X.; Gao, K.; Lu, W.; Zhang, Y.; Dai, L.; Zhao, W.; Xiong, C.; Wang, W.; Liu, Y.; Ni, Y. Facile synthesis of Ag NPs@ MIL-100(Fe)/ guar gum hybrid hydrogel as a versatile photocatalyst for wastewater remediation: Photocatalytic degradation, water/oil separation and bacterial inactivation. *Carbohydr. Polym.* **2020**, *230*, 115642.

19. Li, X.; Semiramoth, N.; Hall, S.; Tafani, V.; Josse, J.; Laurent, F.; Salzano, G.; Foulkes, D.; Brodin, P.; Majlessi, L.; Ghermani, N.; Maurin, G.; Couvreur, P.; Serre, C.; Bernet-Camard, M.; Zhang, J.; Gref, R. Compartmentalized Encapsulation of Two Antibiotics in Porous Nanoparticles: an Efficient Strategy to Treat Intracellular Infections. *Part. Part. Syst. Charact.* **2019**, *36*, 1800360-n/a.

20. Kirakci, K.; Bůžek, D.; Peer, P.; Liška, V.; Mosinger, J.; Křížová, I.; Kloda, M.; Ondrušová, S.; Lang, K.; Demel, J. Polymeric Membranes Containing Iodine-Loaded UiO-66

Nanoparticles as Water-Responsive Antibacterial and Antiviral Surfaces. *ACS Appl. Nano Mater.* **2021**, *5*(1), 1244, .

21. Wang, Z.; Fu, Y.; Kang, Z.; Liu, X.; Chen, N.; Wang, Q.; Tu, Y.; Wang, L.; Song, S.; Ling, D.; Song, H.; Kong, X.; Fan, C. Organelle-Specific Triggered Release of Immunostimulatory Oligonucleotides from Intrinsically Coordinated DNA–Metal–Organic Frameworks with Soluble Exoskeleton. *J. Am. Chem. Soc.* **2017**, *139*(44), 15784, .

22. Jaros, S. W.; Król, J.; Bażanów, B.; Poradowski, D.; Chrószcz, A.; Nesterov, D. S.; Kirillov, A. M.; Smoleński, P. Antiviral, Antibacterial, Antifungal, and Cytotoxic Silver(I) BioMOF Assembled from 1,3,5-Triaza-7-Phoshaadamantane and Pyromellitic Acid. *Molecules* **2020**, *25*(9).

23. Gupta, I.; Farinas, E. T.; Mitra, S. Development of carbon nanotube-metal organic framework (MOF) hybrid antiviral microfiltration membrane. *Sep. Purif. Technol.* **2023**, *315*, 123766.

24. Ejsmont, A.; Warowicka, A.; Broniarczykb, J.; Goscianska, J. The synergistic effect of Cu-MOF nanoparticles and immunomodulatory agent on SARS-CoV-2 inhibition. *Chem. Commun.* **2023**, *59*, 4907-4910.

25. Abdelhameed, R. M.; Hasanin, M. S.; Hashem, A. H. Carboxymethyl cellulose/sulfur-functionalized Ti-based MOF composite: synthesis, characterization, antimicrobial, antiviral and anticancer potentiality. *Discover Nano* **2023**, *18*, 75.

26. Ni, L.; Zhu, Y.; Ma, J.; Wang, Y. Novel strategy for membrane biofouling control in MBR with CdS/MIL-101 modified PVDF membrane by in situ visible light irradiation. *Water Res.* **2021**, *188*, 116554.

27. Hajibabaei, M.; Amini, M. M.; Zendehdel, R.; Nasiri, M. J.; Peymani, A. Synthesis, characterization and antibacterial activity of imidazole-functionalized Ag/MIL-101(Cr). *J. Porous Mater*. **2019**, *26*, 1721-1729.

28. Hajibabaei, M.; Zendehdel, R.; Panjali, Z. Imidazole-Functionalized Ag/MOFs as Promising Scaffolds for Proper Antibacterial Activity and Toxicity Reduction of Ag Nanoparticles. *J. Inorg. Organomet. Polym.* **2020**, *30*, 4622-4626.

29. Saghir, S.; Wang, Y.; Xiao, Z. In situ synthesis of multivariant zeolitic tetrazolate imidazole frameworks (ZTIFs) with uncoordinated N-heteroatom sites for efficient adsorption of antiviral drugs. *J. Cleaner Prod.* **2023**, *414*, 137654.

30. Cao, P.; Wu, X.; Zhang, W.; Zhao, L.; Sun, W.; Tang, Z. Killing Oral Bacteria Using Metal–Organic Frameworks. *Ind. Eng. Chem. Res.* **2020**, *59*(4), 1559, .

31. Jaros, S. W.; Guedes da Silva, M. F. C.; Florek, M.; Smoleński, P.; Pombeiro, A. J. L.; Kirillov, A. M. Silver(I) 1,3,5-Triaza-7-phosphaadamantane Coordination Polymers Driven

by Substituted Glutarate and Malonate Building Blocks: Self-Assembly Synthesis, Structural Features, and Antimicrobial Properties. *Inorg. Chem.* **2016**, *55*, 5886-5894.

32. Jaros, S. W.; Guedes da Silva, M. F. C.; Florek, M.; Oliveira, M. C.; Smoleński, P.; Pombeiro, A. J. L.; Kirillov, A. M. Aliphatic Dicarboxylate Directed Assembly of Silver(I) 1,3,5-Triaza-7-phosphaadamantane Coordination Networks: Topological Versatility and Antimicrobial Activity. *Cryst. Growth Des.* **2014**, *14*, 5408-5417.

33. Kirillov, A. M.; Wieczorek, S. W.; Lis, A.; Guedes da Silva, M. F. C.; Florek, M.; Król, J.; Staroniewicz, Z.; Smoleński, P.; Pombeiro, A. J. L. 1,3,5-Triaza-7-phosphaadamantane-7-oxide (PTA□O): New Diamondoid Building Block for Design of Three-Dimensional Metal–Organic Frameworks. *Cryst. Growth Des.* **2011**, *11*, 2711-2716.

34. Jaros, S. W.; Smoleński, P.; Guedes da Silva, F. C.; Florek, M.; Król, J.; Staroniewicz, Z.; Pombeiro, A. J. L.; Kirillov, A. M. New silver BioMOFs driven by 1,3,5-triaza-7-phosphaadamantane-7-sulfide (PTA=S): synthesis, topological analysis and antimicrobial activity. *CrystEngComm* **2013**, *15*, 8060-8064.

35. Lu, X.; Ye, J.; Zhang, D.; Xie, R.; Bogale, R. F.; Sun, Y.; Zhao, L.; Zhao, Q.; Ning, G. Silver carboxylate metal–organic frameworks with highly antibacterial activity and biocompatibility. *J. Inorg. Biochem.* **2014**, *138*, 114-121.

36. Travlou, N. A.; Algarra, M.; Alcoholado, C.; Cifuentes-Rueda, M.; Labella, A. M.; Lázaro-Martínez, J. M.; Rodríguez-Castellón, E.; Bandosz, T. J. Carbon Quantum Dot Surface-Chemistry-Dependent Ag Release Governs the High Antibacterial Activity of Ag-Metal–Organic Framework Composites. *ACS Appl. Bio Mater.* **2018**, *1*(3), 693, .

37. Firouzjaei, M. D.; Shamsabadi, A. A.; Aktij, S. A.; Seyedpour, S. F.; Sharifian Gh., M.; Rahimpour, A.; Esfahani, M. R.; Ulbricht, M.; Soroush, M. Exploiting Synergetic Effects of Graphene Oxide and a Silver-Based Metal–Organic Framework To Enhance Antifouling and Anti-Biofouling Properties of Thin-Film Nanocomposite Membranes. *ACS Appl. Mater. Interfaces* **2018**, *10*(49), 42967, .

38. Firouzjaei, M. D.; Shamsabadi, A. A.; Sharifian Gh, M.; Rahimpour, A.; Soroush, M. A Novel Nanocomposite with Superior Antibacterial Activity: A Silver-Based Metal Organic Framework Embellished with Graphene Oxide. *Adv. Mater. Interfaces* **2018**, *5*, 1701365-n/a.

39. Seyedpour, S. .; Rahimpour, A.; Najafpour, G. Facile in-situ assembly of silver-based MOFs to surface functionalization of TFC membrane: A novel approach toward long-lasting biofouling mitigation. *J. Membr. Sci.* **2019**, *573*, 257-269.

40. Seyedpour, S. F.; Dadashi Firouzjaei, M.; Rahimpour, A.; Zolghadr, E.; Arabi Shamsabadi, A.; Das, P.; Akbari Afkhami, F.; Sadrzadeh, M.; Tiraferri, A.; Elliott, M. Toward Sustainable Tackling of Biofouling Implications and Improved Performance of TFC FO

Membranes Modified by Ag-MOF Nanorods. *ACS Appl. Mater. Interfaces* **2020**, *12*(34), 38285, .

41. Zirehpour, A.; Rahimpour, A.; Arabi Shamsabadi, A.; Sharifian Gh., M.; Soroush, M. Mitigation of Thin-Film Composite Membrane Biofouling via Immobilizing Nano-Sized Biocidal Reservoirs in the Membrane Active Layer. *Environ. Sci. Technol.* **2017**, *51*(10), 5511, .

42. Ma, S.; Zhang, M.; Nie, J.; Yang, B.; Song, S.; Lu, P. Multifunctional cellulose-based air filters with high loadings of metal–organic frameworks prepared by in situ growth method for gas adsorption and antibacterial applications. *Cellulose* **2018**, *25*, 5999-6010.

43. Pejman, M.; Firouzjaei, M. D.; Aktij, S. A.; Das, P.; Zolghadr, E.; Jafarian, H.; Shamsabadi, A. A.; Elliott, M.; Esfahani, M. R.; Sangermano, M.; Sadrzadeh, M.; Wujcik, E. K.; Rahimpour, A.; Tiraferri, A. Improved antifouling and antibacterial properties of forward osmosis membranes through surface modification with zwitterions and silver-based metal organic frameworks. *J. Membr. Sci.* **2020**, *611*, 118352.

44. Zhang, M.; Wang, G.; Wang, D.; Zheng, Y.; Li, Y.; Meng, W.; Zhang, X.; Du, F.; Lee, S. Ag@MOF-loaded chitosan nanoparticle and polyvinyl alcohol/sodium alginate/chitosan bilayer dressing for wound healing applications. *Int. J. Biol. Macromol.* **2021**, *175*, 481-494.

45. Moradi, E.; Rahimi, R.; Safarifard, V.; Azari, S. A Sonochemically-Synthesized Microporous Metal-Organic Framework for the Rapid and Efficient Ultrasonic-Assisted Removal of Mercury (II) Ions in a Water Solution and a Study of the Antibacterial Activity. *Proceedings* **2019**, *41*, 31.

46. Martín-Betancor, K.; Aguado, S.; Rodea-Palomares, I.; Tamayo-Belda, M.; Leganés, F.; Rosal, R.; Fernández-Piñas, F. Co, Zn and Ag-MOFs evaluation as biocidal materials towards photosynthetic organisms. *Sci. Total Environ.* **2017**, *595*, 547-555.

47. Lu, L.; Hu, C.; Zhu, Y.; Zhang, H.; Li, R.; Xing, Y. Multi-functional finishing of cotton fabrics by water-based layer-by-layer assembly of metal–organic framework. *Cellulose* **2018**, *25*, 4223-4238.

48. Nakhaei, M.; Akhbari, K.; Kalati, M.; Phuruangrat, A. Antibacterial activity of three zinc-terephthalate MOFs and its relation to their structural features. *Inorg. Chim. Acta* **2021**, *522*, 120353.

49. Karimzadeh, Z.; Javanbakht, S.; Namazi, H. Carboxymethylcellulose/MOF-5/Graphene oxide bio-nanocomposite as antibacterial drug nanocarrier agent. *Bioimpacts* **2019**, *9*(1), 5, .

50. Bhardwaj, N.; Pandey, S. K.; Mehta, J.; Bhardwaj, S. K.; Kim, K.; Deep, A. Bioactive nano-metal–organic frameworks as antimicrobials against Gram-positive and Gram-negative bacteria. *Toxicol. Res.* **2018**, *7*(5), 931, .

51. Thakare, S. R.; Ramteke, S. M. Fast and regenerative photocatalyst material for the disinfection of E. coli from water: Silver nano particle anchor on MOF-5. *Catal. Commun.* **2017**, *102*, 21-25.

52. Soltani, S.; Akhbari, K.; Phuruangrat, A. Investigation of effective factors on antibacterial activity of Pillared-Layered MOFs. *J. Mol. Struct.* **2021**, *1225*, 129261.

53. Chen, G.; Yu, Y.; Wu, X.; Wang, G.; Gu, G.; Wang, F.; Ren, J.; Zhang, H.; Zhao, Y. Microfluidic Electrospray Niacin Metal-Organic Frameworks Encapsulated Microcapsules for Wound Healing. *Research* **2019**, *2019*.

54. Arpa Sancet, M. P.; Hanke, M.; Wang, Z.; Bauer, S.; Azucena, C.; Arslan, H. K.; Heinle, M.; Gliemann, H.; Wöll, C.; Rosenhahn, A. Surface anchored metal-organic frameworks as stimulus responsive antifouling coatings. *Biointerphases* **2013**, *8*, 29.

55. Shams, S.; Ahmad, W.; Memon, A. H.; Shams, S.; Wei, Y.; Yuan, Q.; Liang, H. Cu/H3BTC MOF as a potential antibacterial therapeutic agent against Staphylococcus aureus and Escherichia coli. *New J. Chem.* **2020**, *44*, 17671-17678.

56. Singbumrung, K.; Motina, K.; Pisitsak, P.; Chitichotpanya, P.; Wongkasemjit, S.; Inprasit, T. Preparation of Cu-BTC/PVA Fibers with Antibacterial Applications. *Fibers Polym.* **2018**, *19*, 1373-1378.

57. Emam, H. E.; Darwesh, O. M.; Abdelhameed, R. M. In-growth metal organic framework/synthetic hybrids as antimicrobial fabrics and its toxicity. *Colloids Surf., B* **2018**, *165*, 219-228.

58. Can, M.; Demirci, S.; Sunol, A. K.; Sahiner, N. An amino acid, l-Glutamic acid-based metal-organic frameworks and their antibacterial, blood compatibility, biocompatibility, and sensor properties. *Microporous Mesoporous Mater.* **2020**, *309*, 110533.

59. Soltani, S.; Akhbari, K.; Phuruangrat, A. Investigation of effective factors on antibacterial activity of Pillared-Layered MOFs. *J. Mol Struct.* **2021**, *1225*, 129261.

60. Zheng, H.; Wang, D.; Sun, X.; Jiang, S.; Liu, Y.; Zhang, D.; Zhang, L. Surface modified by green synthetic of Cu-MOF-74 to improve the anti-biofouling properties of PVDF membranes. *Chem. Eng. J. (Lausanne, Switz.: 1996)* **2021**, *411*, 128524.

61. Azizabadi, O.; Akbarzadeh, F.; Danshina, S.; Chauhan, N. P. S.; Sargazi, G. An efficient ultrasonic assisted reverse micelle synthesis route for Fe3O4@Cu-MOF/core-shell nanostructures and its antibacterial activities. *J. Solid State Chem.* **2021**, *294*, 121897.

62. Liu, Z.; Ye, J.; Rauf, A.; Zhang, S.; Wang, G.; Shia, S.; Ning, G. A flexible fibrous membrane based on copper(ii) metal–organic framework/poly(lactic acid) composites with superior antibacterial performance. *Biomater. Sci.* **2021**, *9*, 3851-3859.

63. Emam, H. E.; Darwesh, O. M.; Abdelhameed, R. M. Protective Cotton Textiles via Amalgamation of Cross-Linked Zeolitic Imidazole Frameworks. *Ind. Eng. Chem. Res.* **2020**, *59*, 10931-10944.

64. Qian, L.; Lei, D.; Duan, X.; Zhang, S.; Song, W.; Hou, C.; Tang, R. Design and preparation of metal-organic framework papers with enhanced mechanical properties and good antibacterial capacity. *Carbohydr. Polym***.** **2018**, *192*, 44-51.

65. Aguado, S.; Quirós, J.; Canivet, J.; Farrusseng, D.; Boltes, K.; Rosal, R. Antimicrobial activity of cobalt imidazolate metal–organic frameworks. *Chemosphere (Oxford)* **2014**, *113*, 188-192.

66. Quirós, J.; Boltes, K.; Aguado, S.; de Villoria, R. G.; Vilatela, J. J.; Rosal, R. Antimicrobial metal–organic frameworks incorporated into electrospun fibers. *Chem. Eng. J. (Lausanne, Switz.: 1996)* **2015**, *262*, 189-197.

67. Chinthamreddy, A.; Karreddula, R.; Pitchika, G. K.; SurendraBabu, M. S. Synthesis, Characterization of [Co(BDC)(Phen)H2O] and [Co(BDC)(DABCO)] MOFs, π..π Interactions, Hirshfeld Surface Analysis and Biological Activity. *J. Inorg. Organomet. Polym.* **2021**, *31*, 1381-1394.

68. Raju, P.; Ramalingam, T.; Nooruddin, T.; Natarajan, S. In vitro assessment of antimicrobial, antibiofilm and larvicidal activities of bioactive nickel metal organic framework. *J. Drug Delivery Sci. Technol.* **2020**, *56*, 101560.

69. Yang, J.; Wang, C.; Liu, X.; Yin, Y.; Ma, Y.; Gao, Y.; Wang, Y.; Lu, Z.; Song, Y. Gallium–Carbenicillin Framework Coated Defect-Rich Hollow TiO 2 as a Photocatalyzed Oxidative Stress Amplifier against Complex Infections. *Adv. Funct. Mater.* **2020**, *30*, 2004861.

70. Li, W.; Zhou, S.; Gao, S.; Chen, S.; Huang, M.; Cao, R. Spatioselective Fabrication of Highly Effective Antibacterial Layer by Surface-Anchored Discrete Metal-Organic Frameworks. *Adv. Mater. Interfaces* **2014**, *2*(2).

71. Zhuang, W.; Yuan, D.; Li, J.; Luo, Z.; Zhou, H.; Bashir, S.; Liu, J. Highly Potent Bactericidal Activity of Porous Metal-Organic Frameworks. *Adv. Healthcare Mat.* **2012**, *1*, 225-238.

72. Nasser Abdelhamid, H.; Abd-Elmonsef Mahmoud, G.; Sharmoukc, W. A cerium-based MOFzyme with multi-enzyme-like activity for the disruption and inhibition of fungal recolonization. *J. Mater. Chem. B* **2020**, *8*, 7548-7556.

73. Hyoung, J.; Kim, H.; Huh, S.; Kim, Y.; Lee, D. N. Antibacterial activities of Cu-MOFs containing glutarates and bipyridyl ligands. *Dalton Trans.* **2019**, *48*, 8084-8093.

74. Khan, S. A.; Bhat, S. A.; Nami, S. A. A.; Kareem, A.; Nishat, N. Design and development of several polymeric metal–organic frameworks, spectral characterization, and their antimicrobial activity. *C. R. Chim.* **2018**, *21*, 872-879.

75. Tamames-Tabar, C.; Imbuluzqueta, E.; Guillou, N.; Serre, C.; Miller, R.; Elkaïm, E.; Horcajadas, P.; Blanco-Prieto, M. J. A Zn azelate MOF: combining antibacterial effect . *CrystEngComm* **2015**, *17*, 456-462.

76. Quaresma, S.; André, V.; Antunes, A. M. M.; Vilela, S. M. F.; Amariei, G.; Arenas-Vivo, A.; Rosal, R.; Horcajada, P.; Teresa Duarte, M. Novel Antibacterial Azelaic Acid BioMOFs. *Cryst. Growth Des.* **2020**, *20*, 370–382.

77. Abdelhameed, R. M.; Darwesh, O. M.; Rocha, J.; Silva, A. M. S. IRMOF-3 Biological Activity Enhancement by Post-Synthetic Modification. *Eur. J. Inorg. Chem.* **2019**, *2019*(9), 1243, .

78. Mohaghegh, N.; Faraji, M.; Abedini, A. Highly efficient multifunctional Ag/TiO2 nanotubes/Ti plate coated with MIL-88B(Fe) as a photocatalyst, adsorbent, and disinfectant in water treatment. *Appl. Phys. A* **2019**, *125*, 1-10.

79. Liu, Z.; Wang, F.; Rena, J.; Qua, X. A series of MOF/Ce-based nanozymes with dual enzyme-like activity disrupting bio lms and hindering recolonization of bacteria. *Biomaterials* **2019**, *208*, 21-31.

80. Wei, Y.; Chen, C.; Zhai, S.; Tan, M.; Zhao, J.; Zhu, X.; Wang, L.; Liu, Q.; Dai, T. Enrofloxacin/florfenicol loaded cyclodextrin metal-organic-framework for drug delivery and controlled release. *Drug delivery* **2021**, *28*, 372-379.

81. Luo, T.; Shakya, S.; Mittal, P.; Ren, X.; Guo, T.; Bello, M. G.; Wu, L.; Li, H.; Zhu, W.; Regmi, B.; Zhang, J. Co-delivery of superfine nano-silver and solubilized sulfadiazine for enhanced antibacterial functions. *Int. J. Pharm.* **2020**, *584,* 119407.

82. Kathuria, A.; Pauwels, A.; Buntinx, M.; Shin, J.; Harding, T. Inclusion of ethanol in a nano-porous, bio-based metal organic framework. *J. Incl. Phenom. Macrocycl. Chem.* **2019**, *95*, 91-98.

83. Nabipour, H.; Soltani, B.; Ahmadi Nasab, N. Gentamicin Loaded Zn2(bdc)2(dabco) Frameworks as Efficient Materials for Drug Delivery and Antibacterial Activity. *J. Inorg. Organomet. Polym.* **2018**, *28*, 1206-1213.

84. Nabipour, H.; Hossaini Sadr, M.; Rezanejade Bardajee, G. Release behavior, kinetic and antimicrobial study of nalidixic acid from [Zn2(bdc)2(dabco)] metal-organic frameworks. *J. Coord. Chem.* **2017**, *70*, 2771-2784.

85. Duncan, M. J.; Wheatley, P. S.; Coghill, E. M.; Vornholt, S. M.; Warrender, S. J.; Megson, I. L.; Morris, R. E. Antibacterial efficacy from NO-releasing MOF-polymer films. *Mater. Adv.* **2020**, *1*, 259-2519.

86. McKinlay, A. C.; Allan, P. K.; Renouf, C. L.; Duncan, M. J.; Wheatley, P. S.; Warrender, S. J.; Dawson, D.; Ashbrook, S. E.; Gil, B.; Marszalek, B.; Düren, T.; Williams, J. J.; Charrier,

C.; Mercer, D. K.; Teat, S. J.; Morris, R. E. Multirate delivery of multiple therapeutic agents from metal-organic frameworks. *APL Mater.* **2014**, *2*, 124108-124108.

87. Yang, W.; Han, Y.; Li, C.; Zhu, L.; Shi, L.; Tang, W.; Wang, J.; Yue, T.; Li, Z. Shapeable three-dimensional CMC aerogels decorated with Ni/Co-MOF for rapid and highly efficient tetracycline hydrochloride removal. *Chem. Eng. J. (Lausanne, Switz.: 1996)* **2019**, *375*, 122076.

88. Lin, S.; Liu, X.; Tan, L.; Cui, Z.; Yang, X.; Yeung, K. W. K.; Pan, H.; Wu, S. Porous Iron-Carboxylate Metal–Organic Framework: A Novel Bioplatform with Sustained Antibacterial Efficacy and Nontoxicity. *ACS Appl. Mater. Interfaces* **2017**, *9*(22), 19248, .

89. Ghaffar, I.; Imran, M.; Perveen, S.; Kanwal, T.; Saifullah, S.; Bertino, M. F.; Ehrhardt, C. J.; Yadavalli, V. K.; Shah, M. R. Synthesis of chitosan coated metal organic frameworks (MOFs) for increasing vancomycin bactericidal potentials against resistant S. aureus strain. *Mater. Sci. Eng. C* **2019**, *105*, 110111.

90. Xu, W.; Zhuang, H.; Xu, Z.; Huang, M.; Gao, S.; Li, Q.; Zhang, G. Design and Construction of Ag@MOFs Immobilized PVDF Ultrafiltration Membranes with Anti-bacterial and Antifouling Properties. *Adv. Polym. Technol.* **2020**, *2020*, 1-11.

91. Noorian, S. A.; Hemmatinejad, N.; Navarro, J. A. R. Bioactive molecule encapsulation on metal-organic framework via simple mechanochemical method for controlled topical drug delivery systems. *Microporous Mesoporous Mater.* **2020**, *302*, 110199.

92. Lashkari, E.; Wang, H.; Liu, L.; Li, J.; Yam, K. Innovative application of metal-organic frameworks for encapsulation and controlled release of allyl isothiocyanate. *Food Chem.* **2017**, *221*, 926-935.

93. Wang, H.; Lashkari, E.; Lim, H.; Zheng, C.; Emge, t. J.; Gong, Q.; Yamb, K.; Jing, L. The moisture-triggered controlled release of a natural food preservative from a microporous metal–organic framework. *Chem. Commun.* **2016**, *52*, 2129-2132.

94. Zhao, J.; Wei, F.; Xu, W.; Han, X. Enhanced antibacterial performance of gelatin/chitosan film containing capsaicin loaded MOFs for food packaging. *Appl. Surf. Sci.* **2020**, *510*, 145418.

95. Wu, Y.; Luo, Y.; Zhou, B.; Mei, L.; Wang, Q.; Zhang, B. Porous metal-organic framework (MOF) Carrier for incorporation of volatile antimicrobial essential oil. *Food Control* **2019**, *98*, 174-178.
